# What one sees depends on how far the eye has moved

**DOI:** 10.1038/s41467-026-76503-6

**Published:** 2026-08-30

**Authors:** Y. Howard Li, Michele A. Cox, Jonathan D. Victor, Michele Rucci

**Affiliations:** 1 Department of Brain and Cognitive Sciences, University of Rochester, Rochester, NY USA; 2 Center for Visual Science, University of Rochester, Rochester, NY USA; 3 Feil Family Brain and Mind Research Institute, Weill Cornell Medical College, New York, NY USA

**Keywords:** Human behaviour, Oculomotor system

## Abstract

Humans explore visual scenes through frequent, rapid gaze shifts known as saccades. These movements redirect the high-acuity region of the retina toward objects of interest, thus selecting information based on location. Here, we show that saccade amplitude provides a separate and complementary form of selection, effectively filtering visual information by spatial frequency rather than location. Building on analyses of the resulting visual input signals, we examined how pattern visibility was influenced by the size of the preceding saccade. A reduction in saccade amplitude attenuates post-saccadic visual sensitivity in an amplitude-dependent range of low spatial frequencies. This effect is highly robust, so that even small changes in saccade size considerably affect visibility. We show that this phenomenon arises from the way that the magnitude-dependent kinematic characteristics of saccades transform the visual world into a spatiotemporal flow. That is, post-saccadic visibility closely follows theoretical predictions based on the spatial information that saccade transients convey within the temporal bandwidth of retinal sensitivity. Thus, saccades not only guide selection based on location, but also filter visual information based on scale, actively shaping perception.

## Abstract (web summary)

People explore the visual world through rapid eye movements called saccades. Here, the authors show that the size of each saccade determines which level of spatial detail becomes most visible after the movement.

## Introduction

Sensory systems tend to be highly sensitive to changes in their input signals and often leverage these variations to establish sensory representations. This strategy makes sense, as input fluctuations hold significant evolutionary value, providing information not only about object motion—such as the movement of a prey or a predator—but also about the spatial structure of the scene. As an organism moves, even stationary objects generate dynamic sensory signals, offering valuable cues about their location and shape. It is thus not surprising that the use of spatial information derived from self-motion has been widely documented across various sensory modalities, including audition^1,2^, somatosensation^3,4^, and olfaction^5^.

**Fig. 1:**
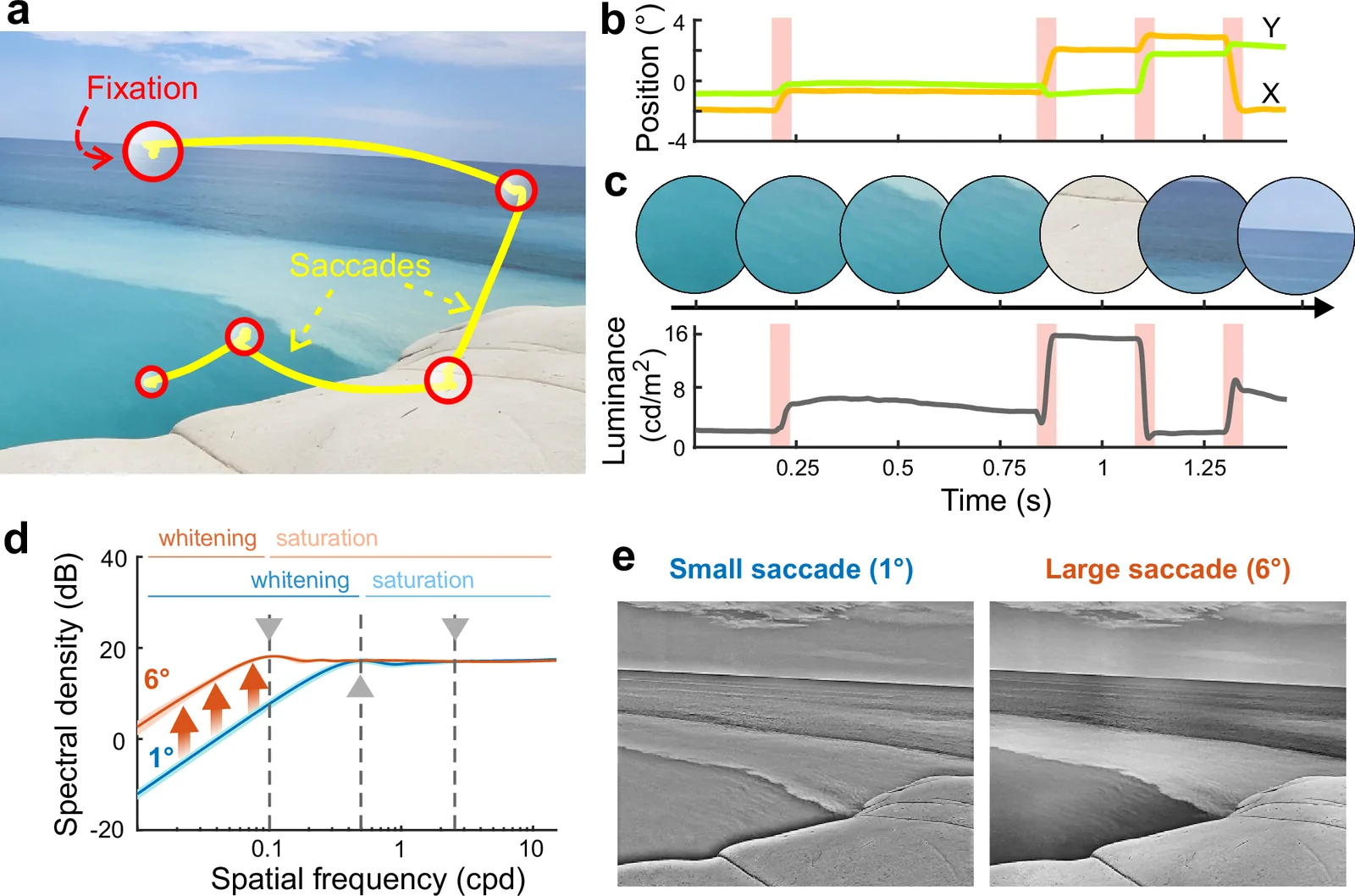
Visual input signals resulting from saccades. **a** Example eye trace superimposed on the observed scene. During natural viewing, saccades (yellow lines) continually alternate with periods of fixation (red circles). **b** The same trace shown as a function of time. Both horizontal (green line) and vertical gaze displacements (orange) are shown. Pink regions mark the periods of saccades. **c** Eye movements modulate input luminance signals. The visual flow experienced by a circular patch on the retina (1^∘^ diameter; top panel) across evenly spaced time points as the eye moves following the trace in *b*. The bottom trace shows the average luminance. Note the abrupt changes introduced by saccades. **d** Retinal input depends on saccade amplitude. Net power of saccade-induced luminance transients within the range of human temporal sensitivity^70^. Data show means across *N *= 9 observers during free-viewing natural scenes  ± one standard deviation (shaded regions). Note the presence of a whitening band in which power increases with spatial frequency before saturating, and in which larger saccades yield stronger luminance modulations. The bandwidth of this whitening region is inversely proportional to saccade amplitude^36^. Gray triangles mark the spatial frequencies used in the experiments of Figs. 2 and 5 (up-pointing: reference, down-pointing: probe). **e** Spatial information delivered in the form of temporally useful luminance modulations by saccades of 1^∘^ and 6^∘^ amplitude when viewing the image in *a*. The two panels show the output of spatial filters whose gains follow the power redistributions shown in *d*, thus representing the strength of the modulations that fall within the temporal range of human sensitivity.

In the visual modality, where two-dimensional space is already explicit on the retina, extraction of spatial information from the temporal changes due to eye or head movement, has been primarily investigated in the context of depth perception, where it provides a powerful cue^6–8^. However, it has long been argued that this encoding strategy may play a more fundamental role in the visual system^9–14^. This is because the visual system responds strongly to input changes^15–19^, and humans incessantly move their eyes, even in the periods in between saccades (Fig. 1a–c), when a slow meandering eye motion, known as ocular drift, continually modulates the visual signals experienced by photoreceptors^20–22^. By transforming a stationary visual scene into a spatio-temporal luminance flow on the retina, ocular drift delivers fine-scale spatial information in the temporal frequency range of highest neuronal sensitivity^23^. Considerable evidence supports the hypothesis that the visual system uses these input fluctuations to encode fine detail, including impairments in pattern discrimination in the absence of fixational motion^24–26^ and a tight coupling between the strength of fixational modulations and visual sensitivity^27,28^.

Although research in this area has primarily focused on the input modulations caused by ocular drifts, several observations suggest that a similar strategy of active space-time encoding applies to the signals resulting from other types of eye movements as well. During normal examination of a visual scene, the most prominent luminance transients arise not from fixational movements, but from saccades^29–33^, which—with their well-defined kinematics^34,35^—yield highly structured signals to the retina. These transients differ from the modulations caused by ocular drifts: since saccades induce larger and faster shifts in the retinal image, they cause pronounced input changes for stimuli at low spatial frequencies^36^, where drifts have little effect. In keeping with this observation, saccades have been found to enhance low-frequency sensitivity relative to equivalent periods of eye drifts^37^. Furthermore, coarse-to-fine processing dynamics^38–41^ have been observed during the typical saccade-drift cycle^42^, as one would expect if the visual system integrates the spatial information conveyed by the temporal signals resulting first from saccades and then from drifts.

The proposal that encoding space via oculomotor-induced transients extends to saccades leads to an unexpected and testable prediction: what one sees after a saccade should depend on how far the eyes have moved. This follows because saccade amplitude is tightly linked to saccade kinematics^34^, which is the primary determinant of the spatial information that saccade transients deliver within the temporal bandwidth of visual sensitivity. As explained in detail below, this prediction is specific: increasing saccade amplitude should selectively enhance visibility within an amplitude-dependent band of low spatial frequencies.

In this study, we directly test this hypothesis by measuring visual performance in human observers after saccades of various amplitudes. We show that saccade amplitude shapes post-saccadic visibility as predicted by the signals that saccade transients deliver within the temporal sensitivity range of the visual system. Thus, saccades select visual information by spatial scale as well as by location.

## Results

Our hypothesis builds on the spatial structure of saccade-induced luminance transients, previously characterized in detail by Mostofi et al. (2020)^36^ and summarized in Fig. 1 (see also Supplementary Fig. S1). Visual input signals from saccades are often approximated as those resulting from a constant-velocity translation of extended duration (the standard uniform-motion proportionality between spatial and temporal frequency), but real saccades are brief ballistic movements with stereotyped, smooth velocity profiles, including acceleration and deceleration phases. These kinematics distribute power over a broad range of temporal frequencies. How much of this redistributed power falls within the temporal bandwidth of visual sensitivity depends jointly on the stimulus spatial frequency and saccade amplitude. For a given saccade, the resulting luminance transients exhibit a characteristic pattern: at very low spatial frequencies, the eye’s displacement produces little change in luminance; as spatial frequency increases, the power of the modulation initially grows approximately with the square of spatial frequency—a “whitening” region in which the induced modulations tend to counterbalance the power spectrum of natural scenes. Beyond a critical, saccade amplitude-dependent, spatial frequency, the modulation power saturates and no longer depends on the stimulus spatial frequency (the “saturation” region; Fig. 1d).

Importantly, the way stimulus power is redistributed varies systematically with saccade amplitude. Because saccade amplitude and speed covary—the so-called main sequence^34^—larger saccades are also faster, and saccades of different sizes can be approximated as spatial-scalings—rather than temporal scalings—of one another^36^. Owing to the inverse relation between scaling in space and spatial frequency (a spatial expansion corresponds to a contraction in frequency), the width of the whitening region is inversely proportional to saccade amplitude. Thus, a small saccade (1°, blue curve in Fig. 1d) exhibits a wider whitening region than a large saccade (6°, orange curve), reaching saturation at a higher cut-off spatial frequency but delivering less power at every spatial frequency within this band. As a consequence, there is a range of spatial frequencies in which larger saccades deliver stronger luminance modulations than smaller ones (red arrows in Fig. 1d).

This interaction between saccade kinematics, spatial frequency, and the resulting temporal modulation implies that, in the temporal domain, a saccade effectively acts as a spatial filter with characteristics determined by saccade amplitude. To provide an intuition, Fig. 1e illustrates the spatial information delivered by a small (1°) and a large (6°) saccade over the image in Fig. 1a. In these panels, the image has been filtered by spatial filters whose gain follows the power redistributions resulting from the two saccades (Fig. 1d), so as to represent the strength of the luminance modulations that fall within the temporal sensitivity range of the visual system. It is evident that transients from saccades of different amplitude provide different spatial information. In this study, we ask whether this difference in input signals translates into a measurable difference in perception.

**Fig. 2:**
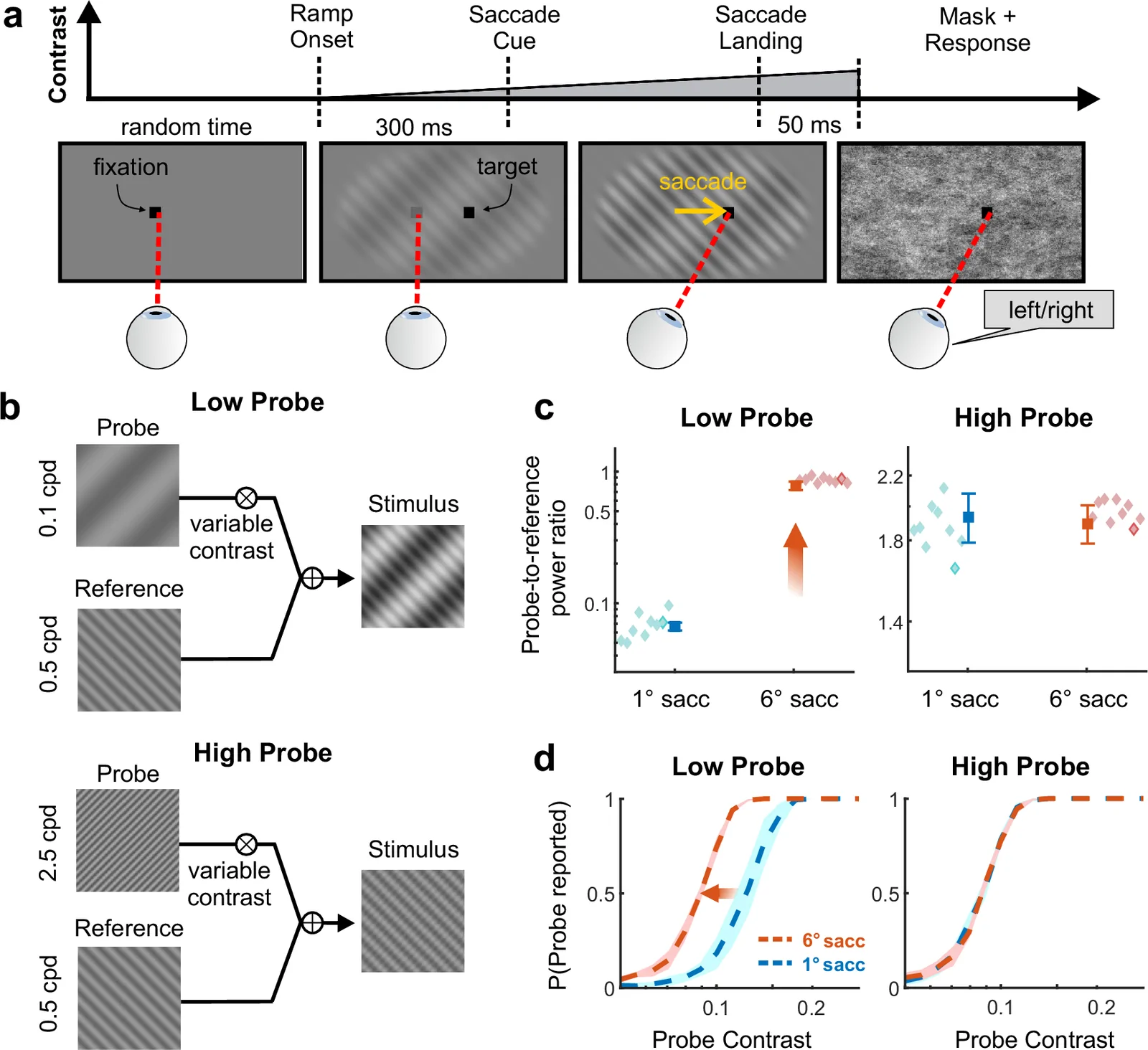
Experimental design and theoretical predictions. **a** Observers made an instructed saccade (amplitude 1^∘^ or 6^∘^) over a stimulus composed of two superimposed orthogonal gratings. The stimulus slowly ramped up in contrast, starting at the beginning of the trial, and was masked 50 ms after the end of the saccade by a Brown noise field (spectral density  ∝ *f*^−2^; *f* spatial frequency). Participants reported which of the two gratings was more visible. **b** One of the gratings (the Reference) was always presented at a fixed contrast, whereas the contrast of the other (the Probe) varied across trials. The spatial frequency of the Probe was either lower (0.1 cpd; Low-Probe condition) or higher (2.5 cpd; High-Probe condition) than the frequency of the Reference (0.5 cpd). **c** Relative strengths of the luminance signals resulting from saccades at the spatial frequencies of the Probe and the Reference. Data points represent the ratio of input power delivered by two equal-contrast gratings at the two frequencies for both small (1^∘^) and large (6^∘^) saccades. Larger saccades emphasize the Probe relative to the Reference in the Low-Probe condition, but not in the High-Probe condition. Diamonds are individual data from the experiment in panel (*a*) (*N* = 9; darker contours mark the observer in Fig. 3a, b). For comparison, squares represent the mean power ratios, averaged across participants (*N* = 9), resulting from saccades with similar amplitudes during free viewing of natural scenes. Error bars are  ± one standard deviation. **d** Sensitivity predicted by the simulated responses of transient neuronal populations tuned to the stimulus frequencies and orientations. In the Low-Probe condition, the stronger signal delivered by the larger saccade is expected to enhance the visibility of the Probe (red arrow). In contrast, no difference in visibility is expected following 1^∘^ or 6^∘^ saccades in the High-Probe condition. Shaded areas represent  ± one standard deviation.

In a forced-choice procedure, participants were asked to report which one of two orthogonal gratings was more visible following saccades of either 1° or 6° amplitude (Fig. 2a). The two gratings were superimposed onto each other. One of them—the “Reference"—was always presented at a fixed contrast and frequency (0.5 cycles per degree; cpd). The other—the “Probe"—varied in both spatial frequency and contrast: it was either at lower (0.1 cpd; Low-Probe condition) or higher (2.5 cpd; High-Probe condition) frequency than the Reference (Fig. 2b), and its contrast varied randomly across trials. We estimated the visibility of the Probe relative to the Reference as a function of the Probe contrast.

The frequencies of the gratings and saccade amplitudes were chosen so that the luminance transients delivered in the Low-Probe and High-Probe conditions would lead to contrasting predictions. The fixed frequency of the Reference was selected so that it fell in the saturation range for both 1° and 6° saccades (upper triangle in Fig. 1d). In contrast, the frequency of the Probe was chosen so that the resulting luminance modulations from small and large saccades would differ in one condition, but not in the other. Specifically, in the High-Probe condition, the 2.5 cpd Probe fell within the saturation ranges of both saccades (right-most upside-down triangle in Fig. 1d), thus yielding similar input signals for small and large saccades. In contrast, in the Low-Probe condition, the 0.1 cpd Probe fell within the whitening range of the 1° saccade and in the saturation range of the 6° saccade (left upside-down triangle in Fig. 1d), thus delivering a stronger input signal for the large saccade. Therefore, whereas the 6° saccade always yields identical transients for the Probe and the Reference, with the 1° saccade, the Probe gives a weaker transient than the Reference in the Low-Probe condition, but not in the High-Probe condition.

We confirmed that the saccades executed in our experiments delivered the expected transients by reconstructing, for each participant and each trial, the spatiotemporal luminance flow experienced on the retina. Specifically, we examined how the recorded saccade trajectory redistributed the energy of equal-contrast gratings at the spatial frequencies of the Probe and the Reference and quantified the effective strength of these modulations by weighting their spectral distributions with the temporal sensitivity function of the human visual system^43^. We then compared the strength of the signals from the Probe and the Reference using their power ratio, which effectively acts as a signal-to-noise ratio (SNR) in this task (Fig. 2c). As expected from the analysis in Fig. 1d, the two saccade amplitudes yielded comparable SNRs in the High-Probe configuration. In contrast, in the Low-Probe condition, the SNR associated with a 1° saccade was approximately one order of magnitude lower than that associated with a 6° saccade (Fig. 2c). These modulations were highly robust with respect to trial-to-trial variability in saccade kinematics (Supplementary Fig. S2) and were similar to those observed with non-instructed saccades. For comparison, the bold squares in Fig. 2c show the average power ratios at the spatial frequencies of interest estimated from a large database of saccades collected while observers freely examined natural scenes^36^. Instructed and free-viewing saccades of comparable amplitudes yielded very similar power ratios.

To provide an intuition of the perceptual consequences of these input signals, we estimated the psychometric functions predicted by a simple ideal-observer model (Fig. 2d). In each trial, the model estimated the average responses of simulated V1 neurons to reconstructions of the saccade-induced visual transient experienced by the observers’ retinas. Neurons were tuned to the two orientations and frequencies of the Probe and the Reference, and the model reported as more visible the grating that elicited a higher overall response. Figure 2d shows the model’s probability of reporting the Probe to be more visible than the Reference as a function of the Probe’s contrast. As shown by these data, small and large saccades led to distinct perceptual consequences in the Low-Probe, but not in the High-Probe, condition. In keeping with the strength of the visual input signals, the large 6° saccade improved visibility of the low-frequency probe, as indicated by the leftward shift in the predicted psychometric function (Fig. 2d, left panel). In contrast, with a high spatial frequency Probe, the two saccade amplitudes gave virtually identical psychometric functions, predicting similar visibility (Fig. 2d, right panel).

**Fig. 3:**
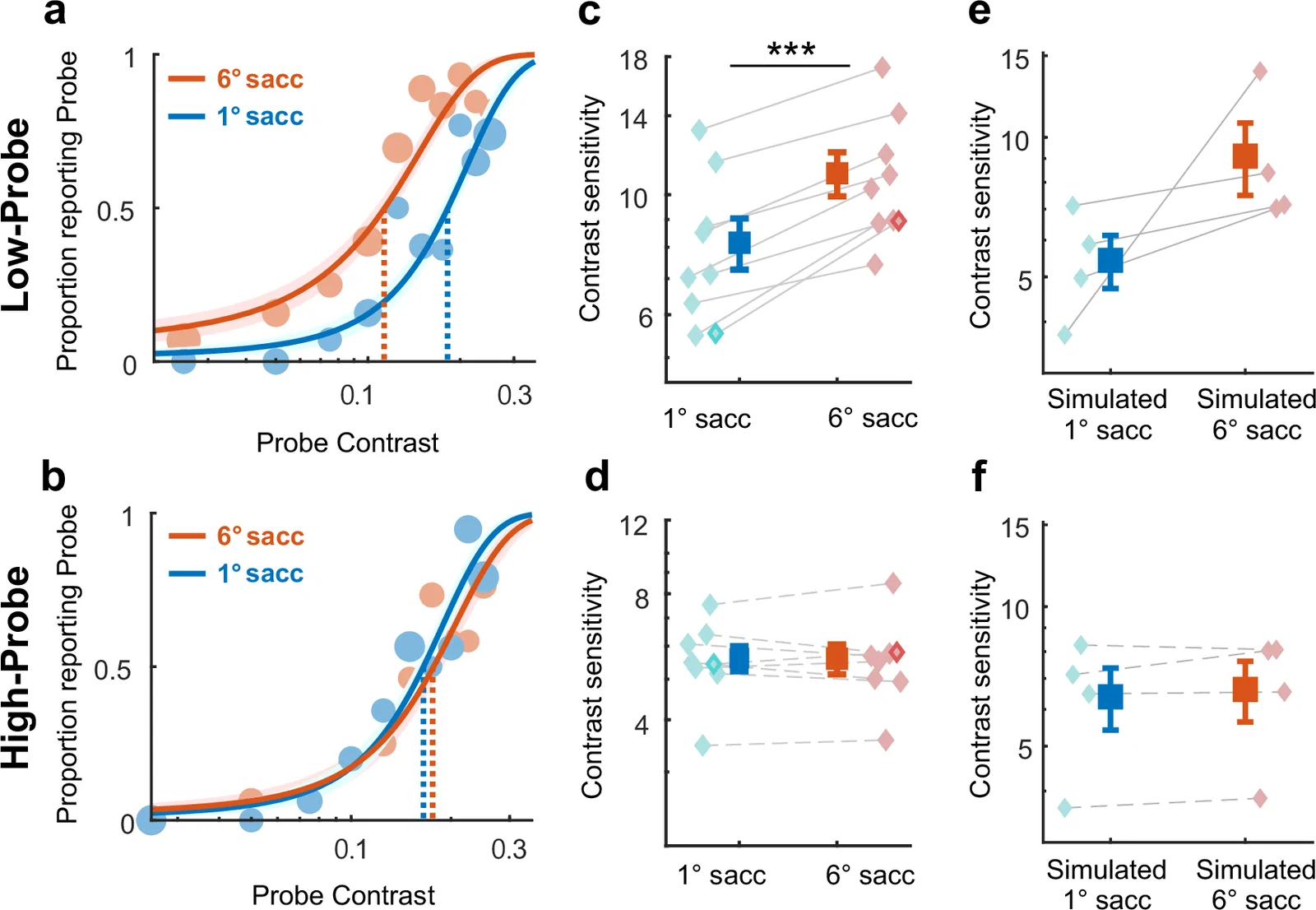
Experimental results. **a, b** Measured psychometric functions for an individual observer. Circles show the probability of reporting the Probe as more visible as a function of its contrast for small (1^∘^, blue) and large (6^∘^, red) saccades; circle size is proportional to the number of trials, and solid curves show fitted psychometric functions. Dashed lines mark the point of subjective equality (the 50% threshold). Shaded areas represent  ± one standard error. **a** Large saccades enhance Probe visibility when the Probe is at a lower spatial frequency than the Reference (*p* < 0.001; one-sided parametric bootstrap test). **b** In contrast, saccade amplitude has no influence when the Probe is at a higher spatial frequency than the Reference (*p* = 0.69; one-sided parametric bootstrap test). **c**, **d** Contrast sensitivity for small and large saccades. A 6^∘^ saccade enhances visibility in the Low-Probe condition (panel **c**, ***p < 0.001, two-sided paired *t*-test) but not in the High-Probe condition (**d**, t(7) = -0.066, *p* = 0.949; two-sided paired *t*-test). Error bars represent standard errors. Diamonds mark individual-observer data; symbols with darker outlines correspond to the participant shown in **a**, **b**. **e**, **f** Sensitivity measured following simulated saccades with Low-Probe (**e**) and High-Probe (**f**) stimuli. In panels **c**–**f**, bold symbols are averages across participants (*N* = 9 in **c**, 8 in **d**, and 4 in **e**, **f**); error bars represent  ± one standard error; solid lines indicate statistically significant effects in individual observers (*p* < 0.01, one-sided parametric bootstrap test).

Perceptual performances measured in our experiments closely followed these theoretical predictions. Figure 3a reports the psychometric functions obtained from one representative observer. In the Low-Probe condition, when the strength of the luminance modulation at the Probe frequency varied with saccade amplitude, the visibility of the Probe was enhanced by a larger saccade, as shown by the leftward shift in the 6° psychometric function relative to the 1° in Fig. 3a. This effect was highly consistent across observers, with all individual participants exhibiting a statistically significant improvement in sensitivity (solid lines in Fig. 3c; *p* < 0.03, one-sided parametric bootstrapping test). On average across observers, contrast sensitivity increased by 37% with the larger saccade (Fig. 3c; t(8) = 9.097, *p* < 0.001, 95% confidence intervals = [2.09, 3.51], Cohen’s *d* = 0.98; two-sided paired *t*-test)). In contrast, in the High-Probe condition, performance was virtually unaffected by the amplitude of the saccade: the psychometric functions measured with 1° and 6° saccades largely overlapped with each other (Fig. 3b), resulting in similar Probe sensitivity in the two conditions (Fig. 3d; t(7) =−0.066, *p* = 0.949, 95% CI = [−0.45, 0.42], Cohen’s *d* = − 0.01; two-sided paired *t*-test).

These perceptual effects were highly robust with respect to possible differences in motor behavior between the Low- and High-Probe conditions. Specifically, the results were not influenced by small differences in saccade reaction times (approximately 50 ms longer for 1° saccades), as demonstrated in Fig. S3 by restricting the analysis to pools of trials with comparable reaction times for both amplitudes. Results were also not affected by the small but measurable variations in saccade directions across conditions (Fig. S4). We found no systematic bias in saccade direction as a function of the orientation of the perceptually more visible grating (Fig. S4c), and the pattern of results remained virtually unchanged when considering only trials in which saccades were oriented above or below the horizontal meridian (Fig. S4d, e).

Importantly, similar results were also obtained with passive exposure to saccade motion, a condition that eliminates possible extra-retinal influences (Fig. 3e, f). In this experiment, rather than executing saccades, observers maintained fixation while the stimulus moved on the display following previously recorded saccade traces. These simulated saccades delivered the same retinal motion as real ones, but lacked associated extra-retinal signals, as no motor command was generated. Yet, as with real saccades, the larger simulated saccade selectively improved visibility at low spatial frequencies: for each individual participant, performance was significantly better with the larger displacement in the Low-Probe condition (*p* < 0.012; one-sided parametric bootstrap test), whereas it remained unchanged following small and large displacements in the High-Probe condition. These results provide further support for the conclusion that the perceptual effects were driven by the retinal input modulations.

To precisely control the strength of luminance modulations, the experiments in Fig. 3 were conducted under tightly controlled conditions, with predictable saccades and finely structured stimuli that juxtaposed gratings at specifically selected frequencies. Outside the laboratory, however, saccades vary in both direction and amplitude, and naturalistic stimuli contain mixtures of spatial frequencies. To examine how our findings extend to more complex and naturalistic conditions, we conducted two additional experiments.

First, we asked whether the effects reported in Fig. 3 persisted when the Probe and the Reference were defined by broader spatial-frequency bands rather than single gratings, and when saccades varied randomly in both amplitude and direction. In keeping with the previous results, the amplitude of the preceding saccade affected stimulus visibility in the Low-Probe but not the High-Probe condition: larger saccades increased the proportion of trials in which observers reported the Probe as more visible only when it contained lower spatial frequencies than the Reference (Supplementary Fig. S5). A repeated-measures ANOVA confirmed a significant interaction between saccade amplitude and spatial frequency (F(1,3) = 36.53, *p* = 0.009, interaction effect = 0.125, 95% CI = [−0.022, 0.271]; two-way repeated-measures ANOVA).

**Fig. 4:**
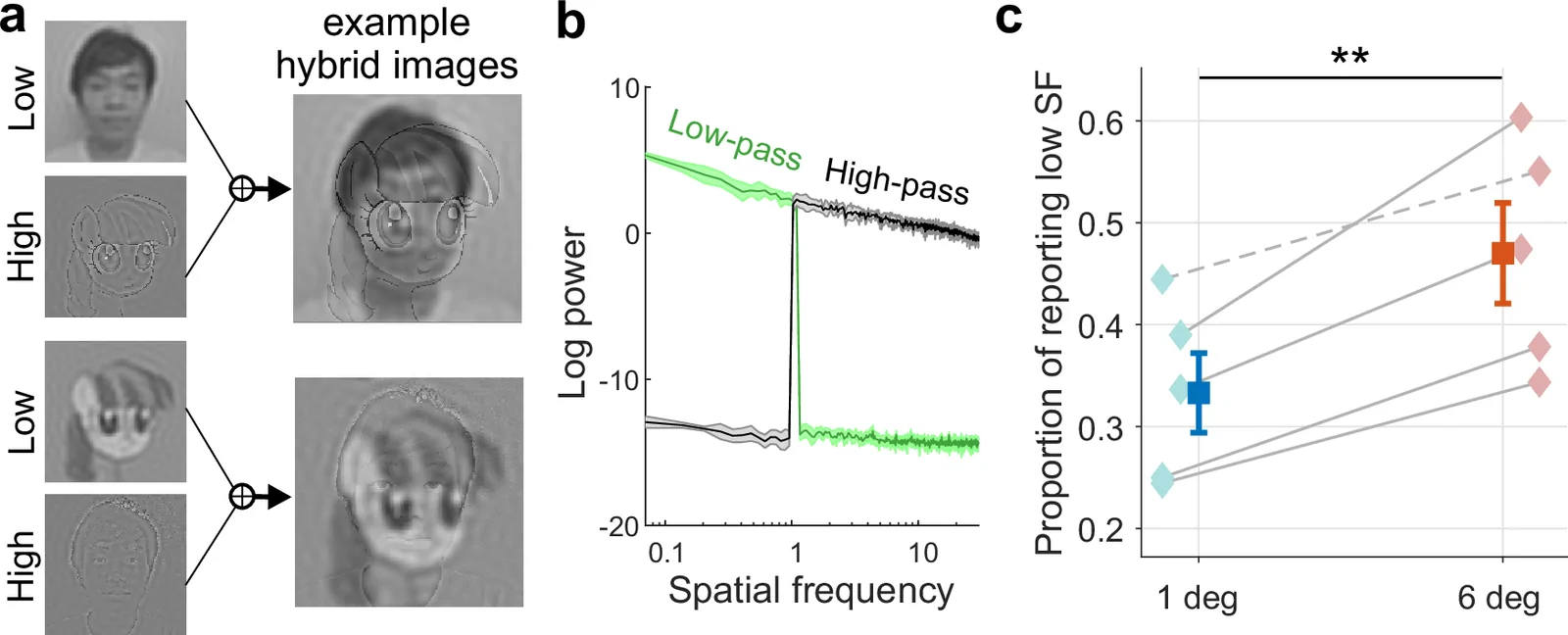
Post-saccadic influence on the visibility of naturalistic stimuli. **a** Participants reported which component of a hybrid image—the low- or the high-frequency component–was more visible following 1^∘^ and 6^∘^ saccades. Stimuli were hybrid images obtained by superimposing high- and low-pass filtered images of human and cartoon faces^44^. The first author, shown in this example image, consented to publication. **b** Amplitude of the stimulus components in the spatial-frequency domain. The green trace shows the low-pass component and the black trace the high-pass component; the cutoff frequency was 1 cpd. **c** Consistent with the stronger luminance modulations delivered within their whitening range, larger saccades enhanced the visibility of the low-frequency component of the stimulus (**p = 0.002; two-sided paired t-test). Diamonds are data from the *N*=5 individual observers. Graphic conventions are as in Fig. 3c.

Next, we examined whether larger saccades would similarly boost the visibility of low spatial frequencies in naturalistic images. To this end, we used hybrid images, constructed by combining the low-spatial-frequency spectrum of one image with the high-spatial-frequency spectrum of another (Fig. 4a–c). Specifically, we used a dataset of hybrid images in which human faces were combined with cartoon faces^44^. Using a procedure similar to that of the experiment in Fig. 2, observers were asked to report which of the two faces appeared more visible after making small or large saccades. Again, consistent with the prediction that larger saccades introduce higher power within the low-spatial-frequency range, participants were more likely to perceive the low-spatial-frequency image when making larger saccades (Fig. 4c, t(4) = 7.520, *p* = 0.002, 95% CI = [0.10, 0.21], Cohen’s *d* = 1.24; two-sided paired *t*-test). These findings show that the amplitude-dependent effects revealed by the experiments in Fig. 3 extends to broadband, naturalistic stimuli.

**Fig. 5:**
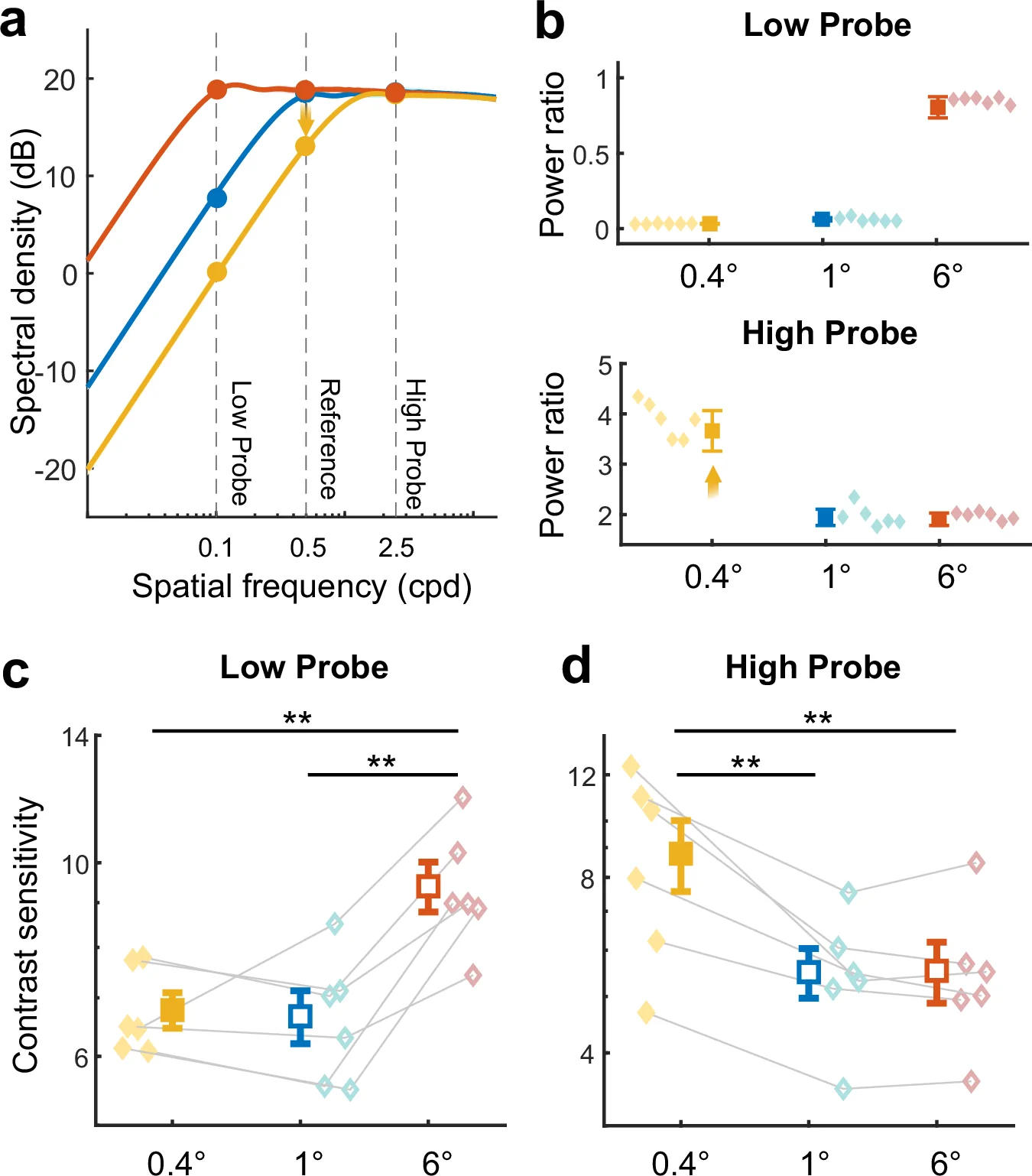
Perceptual reports closely follow the structure of saccade transients. A saccade smaller than 1^∘^ is expected to yield a perceptual improvement in the High-Probe condition. **a** The stimulus power delivered from the previously tested saccades (1^∘^ and 6^∘^) is here compared to the power resulting from a saccade with 0.4^∘^ amplitude (yellow; *cf*. Figure 1d). The smaller saccade extends the region of difference in power, with reduced power delivered by the Reference (yellow arrow). **b** Ratio of power delivered by the Probe and reference. A 0.4^∘^ saccade enhances the signal resulting from the Probe in the High-Probe condition (yellow arrow), when the Probe is at higher spatial frequency than the Reference, but not in the Low-Probe condition. Graphic conventions are as in Fig. 2c. **c, d** Sensitivity measured following saccades with the three amplitudes. In the Low-Probe condition (left), a 6^∘^ saccade improves Probe visibility compared to the 1^∘^ and 0.4^∘^ saccades. In the High-Probe condition (right), a 0.4^∘^ saccade selectively improves visibility compared to both 1^∘^ and 6^∘^ saccades (**p < 0.005). Graphic conventions are as in Fig. 3c. Open symbols represent data points replotted from Fig. 3 for the *N* = 6 participants in this experiment.

To examine how closely perceptual enhancements follow the spatial information contained in saccade transients, we tested a second prediction that follows from the amplitude-dependent specificity of spatiotemporal reformatting. The transients from saccades of different amplitudes form a continuum, with the bandwidth of the whitening region increasing as saccade amplitude decreases^36^. In the experiment of Fig. 3, the Reference and the high-frequency Probe remained in the saturation region for both saccade amplitudes and, therefore, delivered signals of approximately equal strength. However, further decreasing saccade amplitude extends the whitening region to encompass the Reference, weakening the Reference transient by a similar factor to the low-frequency Probe, while leaving the transient from the high-frequency Probe largely unchanged (Fig. 5a). Consequently, a saccade smaller than 1° is expected to exert the opposite influence of that observed in Fig. 3 for larger saccades, enhancing visibility only in the High-Probe condition, i.e., when the Probe is at a higher—rather than lower—spatial frequency than the Reference (Fig. 5b).

To test this prediction, we assessed the visibility of the Probe following a small saccade of 0.4° amplitude. Figure 5c compares these new data to the visibility measurements obtained with the larger saccades 1° and 6°, replotted here from Fig. 3 for the subset of observers who also participated in this second experiment. In the Low-Probe condition, the condition in which a large saccade is beneficial, performance with 0.4° and 1° saccades was virtually identical, and the 6° saccade improved Probe visibility compared to both the 1° saccade (*t*(5) = 6.751, *p* = 0.001, 95% CI = [1.69, 3.77], Cohen’s *d* = 2.04; two-sided paired *t*-test) and the 0.4° saccade (*t*(5) = 4.013, *p* = 0.010, 95% CI = [0.94, 4.30], Cohen’s *d* = 2.18; two-sided paired *t*-test).

Importantly, however, as predicted by the strength of saccade transients, the 0.4° saccade was beneficial in the High-Probe condition, significantly improving the visibility of the Probe when it was at higher frequency than the Reference. This perceptual benefit was substantial relative to both the 1° saccade (*t*(5) = 3.561, *p* = 0.016, 95% CI = [0.91, 5.66], Cohen’s *d* = 1.42; two-sided paired *t*-test) and the 6° saccade (*t*(5) = 3.600, *p* = 0.016, 95% CI = [0.93, 5.58], Cohen’s *d* = 1.35; two-sided paired *t*-test). The effect was also highly consistent across observers: all participants exhibited significantly higher performance with a 0.4° saccade than a 1° saccade (*p*  < 0.002, one-sided bootstrap tests). This effect is remarkable given that it occurs with a mere half-degree difference in saccade amplitude. It shows that even small changes in eye movements carry important consequences for visual functions.

## Discussion

Humans explore visual scenes by making saccades of various amplitudes. These eye movements serve the primary function of redirecting the high-acuity fovea toward objects of interest, but in doing so they also deliver strong luminance transients to the retina. Previous analyses of the spatiotemporal structure of retinal input signals have shown that the spatial information conveyed by these transients varies systematically with saccade amplitude^36^, raising the hypothesis that visual perception may also change following saccades of different sizes. Here we directly tested these predictions at the perceptual level and found robust, highly consistent changes in spatial sensitivity as a function of saccade amplitude. In line with the saccade-dependent reformatting of visual input, increasing saccade amplitude selectively enhanced visibility in a low range of spatial frequencies while narrowing the bandwidth over which this enhancement occurred. Conversely, progressively smaller saccades increased the relative visibility of higher spatial frequencies by attenuating power at low frequencies. These findings demonstrate that saccades do more than simply select where information is sampled; they simultaneously filter what spatial information is encoded through their amplitude-dependent transients.

To quantitatively link perceptual sensitivity to the structure of saccade-induced transients, the experiments of Figs. 2, 3 and 5 used controlled saccade amplitudes and tailored stimuli that contrasted gratings at selected spatial frequencies. These controlled experiments allowed us to unambiguously test the resulting predictions and demonstrate that post-saccadic visibility closely tracks the way saccades of different sizes redistribute spatial information in the temporal domain—specifically, the relative strength of the input modulations across spatial frequencies within the temporal bandwidth of visual sensitivity. Importantly, the underlying mechanism is general: the spatiotemporal reformatting of retinal input varies continuously with saccade amplitude, and during natural viewing it progressively amplifies and equalizes luminance modulations over a narrower low-spatial-frequency band as saccade amplitude increases. A direct consequence is that the spatial scale of the earliest post-saccadic analysis—the “gist” of the visual scene extracted immediately after a gaze shift—depends on the amplitude of the preceding saccade, with larger saccades promoting a coarser initial representation. This effect is evident in the hybrid-image experiment (Fig. 4), where larger saccades selectively enhanced the visibility of the low-spatial-frequency component of naturalistic scenes.

This dependence of visibility on saccadic transients is both substantial and highly robust. As shown by our data, sensitivity changes by approximately 40% following saccades with amplitudes of 1° and 6° (Fig. 3). While this perceptual effect is attenuated relative to the difference in input power between the corresponding retinal input transients (nearly an order of magnitude; Fig. 1d), such attenuation is expected given the well-established nonlinear relationship between luminance and brightness^45–47^, as well as the mechanisms of contrast gain control^48,49^ and divisive normalization^50,51^ identified in early visual pathways. Consistent with this interpretation, incorporating a compressive nonlinearity to approximate normalization in our model was sufficient to quantitatively reproduce the observed perceptual effects (Fig. 2d). Importantly, saccade-induced perceptual enhancements are consistent across observers, persist despite trial-to-trial variability in saccade kinematics, and generalize to naturalistic stimuli and to conditions in which saccade direction and amplitude vary unpredictably. Moreover, similar enhancements were measured when observers maintained fixation while exposed to spatiotemporal signals mimicking those delivered by saccades (Fig. 3e,f), a condition that eliminates extra-retinal influences and isolates the contribution of retinal input, supporting our conclusion that these perceptual reports are driven by the spatial information conveyed by luminance transients.

Previous studies have reported various types of visual enhancements immediately following saccades. For instance, both contrast sensitivity and pattern discriminability improve in the short period after saccade landing^52–54^. Similarly, ocular-following responses—the tracking of a suddenly moving object—are facilitated when motion onset occurs right after a saccade rather than during fixation^55–57^. Perceptual modulations around saccades have often been linked to extra-retinal processes related to attention^58–60^, saccade planning^61^, and execution^62,63^. Our results do not contradict these findings, but rather add to the body of literature showing that the visual input signals resulting from saccades also play an important role^64,65^: luminance changes introduced by saccades have been implicated in ocular following^57,66^, visual stability^67,68^, and contrast sensitivity^37,42^. In the context of contrast sensitivity, rapid gaze shifts have been shown to be specifically beneficial for the visibility of low spatial frequencies, consistent with the stronger signals that saccades deliver relative to fixational drifts in this frequency range^42^. Our results go beyond this literature by showing that post-saccadic visibility depends systematically on saccade amplitude, as predicted by the amplitude-dependent spatiotemporal reformatting of retinal input.

The results of this study extend encoding strategies previously reported for fixational eye movements. During fixation, ocular drift continually shifts the retinal image, generating luminance modulations that amplify higher spatial frequencies up to a critical frequency that is inversely proportional to the drift diffusion constant^26^. During viewing of natural scenes, this redistribution equalizes (whitens) the power at nonzero temporal frequencies over a band wider than the whitening range of saccades^23^. Consistent with this input reformatting, sensitivity to high spatial frequencies is selectively impaired when drift-induced retinal motion is eliminated^24,25^. Spatial sensitivity also closely follows the strength of the resulting modulations^28^, and individual drift characteristics predict acuity limits^27^. Moreover, humans tune drift according to task demands in a manner that enhances these modulations^26,69^. By showing that a similar encoding principle extends to saccade-induced transients, the present study supports a general strategy of active visuomotor encoding: visual representations depend not only on the image on the retina, but also on how eye movements shape the incoming flow of visual information.

It is important to emphasize that the theoretical predictions motivating our experiments are based on the full saccade waveform, not on extrapolations from instantaneous saccade speed at any given moment. The common intuition that saccade-induced temporal frequency is approximated by the product of saccade velocity and stimulus spatial frequency effectively treats the saccade as equivalent to uniform motion, that is, a constant-velocity translation of infinite duration. Real saccades, by contrast, start and end at zero velocity, generating state transitions in the retinal input and redistributing stimulus power across a broad range of temporal frequencies. To correctly estimate the power spectrum of these transients, we analyzed the luminance signals resulting from the full saccade waveform in their natural context, embedding them within periods of constant luminance. By attenuating spectral leakage and enabling proper handling of boundary discontinuities, this approach preserves the spectral content of the saccade transient and its functional interpretation: each point in the maps of Fig. S1e is proportional to the response strength elicited in a linear filter tuned to the corresponding spatial and temporal frequencies. This in turn enables estimation of the strength of the saccade-elicited visual input that falls within the bandwidth of human temporal sensitivity^43,70^ and drives standard models of early visual neurons^71,72^. More generally, analyses of the spatiotemporal input on the retina may also help explain results obtained in paradigms involving saccades and dynamic stimuli, including stimuli presented with saccade-like temporal profiles and perceptual judgments of saccade-induced smear^73,74^.

The post-saccadic enhancements observed here raise interesting hypotheses. One immediate question is whether saccade-induced transients contribute to saccadic suppression—the reduced sensitivity to probes briefly displayed during saccades relative to fixation^53,75–77^. It has long been argued that saccadic suppression involves both extra-retinal signals^78,79^ and retinal factors^80–82^. Suppression is observed even when saccades are made across uniform backgrounds or in complete darkness^78^, but it is more pronounced when saccades are executed over structured backgrounds^77,83^. Our results suggest a possible contribution to the visually driven retinal component when saccades are made over a structured background (Supplementary Fig. S7). Under these conditions, saccade-induced transients may act as masking noise relative to the *signal* generated by the probe flash: whereas oculomotor-induced luminance modulations vary across spatial frequencies (Fig. 1d), a brief flash distributes temporal power uniformly at different spatial frequencies. Consequently, because saccades and ocular drift yield comparable power at high spatial frequencies, the signal-to-noise ratio (SNR) for a high-frequency probe is similar whether it is presented during a saccade or at fixation. In contrast, the SNR for a low-frequency probe is substantially reduced during saccades (relative to drift), because saccades generate considerably more low-frequency background power than drift. Larger saccades are expected to further amplify this effect by producing stronger background transients in this frequency range. These predicted SNR changes are consistent with the classical finding that saccadic suppression is strongest at low spatial frequencies^76^, as well as with reports of amplitude-dependent suppression effects^84^.

A second emerging question is whether the perceptual consequences of saccade transients also influence saccade planning. Since saccade amplitude acts as a perceptual spatial filter, saccade planning might incorporate not only selection in space by placing the fovea on locations of interest, but also selection in spatial frequency, by adjusting saccade amplitude to enhance target sensitivity or reduce interference from distractors. Although dedicated experiments will be required to disentangle such effects from the benefits of foveation, several observations are consistent with the idea that saccade control serves functional roles beyond foveal placement. Visual processing is not suspended during saccades, and information can be extracted from intra-saccadic retinal input^42,67,74,85–87^. Moreover, saccades occur in species without a fovea^88,89^. In humans, saccade amplitude is modulated by stimulus content and task demands in ways compatible with the effects reported here: amplitudes increase when searching for low-spatial-frequency targets^90^ and when viewing low-pass filtered images relative to high-pass filtered images^91–93^. In addition, impaired foveal function is often accompanied by increased saccade amplitude^94^.

Finally, our results indicate that altered control of saccade amplitude is expected to carry perceptual consequences by changing saccade transients. Abnormal saccades have been reported in multiple conditions that also present visual impairments. For example, the faster and smaller saccades observed in schizophrenia^95–98^ may alter the normal coarse-to-fine perceptual dynamics associated with the saccade-fixation cycle^42^, potentially contributing to reported deficits in figure-ground segmentation and visual acuity^99,100^. Similarly, the slower saccades observed in some individuals on the autism spectrum^101^ would be expected to further weaken transients in the whitening band, a change that could contribute to reduced sensitivity at low spatial frequencies^102^. These considerations emphasize the need to study visual impairments in conjunction with eye movements^103,104^.

## Methods

### Participants

Data reported in this article come from a total of 21 participants (12 males and 9 females; age range 19–36). Twelve of them (7 males and 5 females; age range 19–36) took part in the experiments described in this study. Specifically, 9 (5 males and 4 females; age range 19–36) participated in the main instructed-saccade experiment with 1° and 6° saccades (*N*=9 in Fig. 3c; *N*=8 in Fig. 3d, as one participant did not complete the high-probe condition). Six of these (3 males and 3 females; age range 19–27) also completed the 0.4° amplitude condition (Fig. 5). Five participants (4 males and 1 female; age range 25–32) took part in the hybrid-images experiment (Fig. 4), including 2 who also completed the main experiment. Four of these (3 males and 1 female, including the 2 from the main experiment; age range 25–32) also participated in both the simulated-saccade experiment (Fig. 3e, f) and the broadband-stimuli experiment with randomized saccade direction (Fig. S5). In addition, oculomotor data from 9 additional observers (5 males and 4 females; age range 21–31), previously collected during free-viewing of natural scenes^36^, were used for the spectral analyses (Figs. 1d, 5a, S1d) and as the free-viewing reference in Figs. 2c and 5b.

All participants had 20/20 or better uncorrected visual acuity as determined by a standard Snellen eye chart. With the exception of one author, all were naïve to the purpose of the experiments and were compensated for their participation. Approval from the Research Subjects Review Board at the University of Rochester was obtained for all reported experiments, and informed consent was obtained from all participants.

Subject numbers were chosen to support binomial inference (*p* < 0.005) about whether effects that were statistically significant in individual observers generalized to a majority of the broader population^105,106^. Sex and/or gender was determined by self-report and were not considered in the study design, as reported sex-related differences in saccade dynamics are on the order of 5%^107^.

### Stimuli and apparatus

Stimuli were displayed on a LCD monitor (ASUS258) at a resolution of 1920  × 1080 pixels and at a refresh rate of 200 Hz. They were rendered by means of EyeRIS^108^, a custom system for gaze-contingent display control designed to guarantee precise synchronization between the stimulus on the monitor and oculomotor recordings. Stimuli were viewed binocularly.

The experiments of Figs. 3, 5, and Supplementary Fig. S5 focused on the low spatial-frequency range that predominates in natural scenes and in which changes in saccade amplitude have the strongest influence on saccade-induced transients. In Figs. 3 and 5, stimuli consisted of two superimposed, orthogonal sine-wave gratings tilted by  ± 45° relative to the horizontal meridian. One of the gratings (the ‘Reference”) had a fixed spatial frequency of 0.5 cpd and a fixed Michelson contrast of 0.1. The other grating (the ‘Probe”) was either lower (0.1 cpd; Low-Probe condition) or higher (2.5 cpd; High-Probe condition) in spatial frequency than the Reference, and its contrast varied randomly across trials (0.01–0.4; method of constant stimuli). In Fig. S5, the individual gratings were replaced by oriented noise patterns obtained by filtering white noise at the same center frequencies (0.1, 0.5, or 2.5 cpd) with a bandwidth of  ± 1 octave and an orientation bandwidth of  ± 5°. Stimuli were presented over a uniform gray background (9.1 *cd*/*m*^2^) and were fully visible through an elliptical window (15° by 8°) surrounded by a region of decreasing transparency (Gaussian envelope with *σ* = 2°). Low- and High-Probe trials were intermixed.

In the experiment of Fig. 4, stimuli consisted of hybrid images constructed by combining human and cartoon faces such that the low-spatial-frequency component (below 1 cpd) was taken from one image and the high-spatial-frequency component (above 1 cpd) from the other. Source images were synthetically generated using a StyleGAN generative adversarial network^44^. For each stimulus, the assignment of human versus cartoon images to the low- or high-pass component was randomized.

The movements of the right eye were continuously recorded by means of a digital Dual Purkinje Image eye-tracker (the dDPI^109^), a system capable of resolving movements smaller than 1 arcminute. Data were acquired in digital form at 1 kHz. To ensure precise measurement of eye movements, participants’ heads were immobilized with a custom dental-imprint bite bar and a headrest. The monitor was located at a distance of 83 cm from the observer, resulting in each pixel subtending an angle of approximately $1.{2}^{{\prime} }$.

### Procedure

Participants performed multiple experimental sessions, each lasting approximately one hour. Data were collected in blocks of 100 trials (~10 minutes per block), with rest periods in between. Before each block of trials, a gaze-contingent calibration procedure ensured accurate localization of the line of sight. This calibration, described in detail in previous publications^110^ consisted of two phases: a standard oculomotor calibration followed by a gaze-contingent refinement. In the refinement phase, observers fixated again on the nine points of a 3  × 3 grid and corrected any offsets to the estimated gaze location, which was displayed in real-time on the display. These corrections were then incorporated into a bilinear transformation that mapped oculomotor measurements into screen coordinates.

A trial began with the observer maintaining fixation within 1° of the marker (a $1{0}^{{\prime} }$ dot) for at least 1 s. The stimulus then started ramping up in contrast, reaching its maximum after 1 s. A shift in the fixation marker served as a saccade cue, instructing the participant to perform a saccade of a specific amplitude. The saccade cue occurred 300 ms after ramp onset, an interval chosen to ensure that the saccade would deliver a sufficiently strong transient to the retina. With the exception of the broad-band stimulus experiment (Fig. S5), in which saccade amplitude and direction varied randomly across trials, participants performed horizontal saccades of a given amplitude within each block of trials. In the experiments of Figs. 3 and 5, blocks either alternated between two saccade amplitudes (1° and 6°; *N* = 9 participants) or among three amplitudes (0. 4°, 1°, and 6°; *N* = 6, a subset of the 9). In the experiment of Fig. S5, both saccade amplitude (1° or 6°) and direction (up, down, left, or right) varied pseudo-randomly from trial to trial, with target locations selected so as to remain within the central 12° area of the display. Saccades with these amplitudes are common in everyday viewing: saccades smaller than 1° are predominant during high-acuity inspection^54,110,111^, whereas saccades of 1° and above occur routinely during scene exploration, visual search, and reading^22^.

Eye velocity was monitored continuously in real time by EyeRIS, and saccade landing was defined as the time at which eye speed dropped below 3°/s. The stimulus remained visible for 50 ms after saccade landing before being masked by a Brown noise field covering the entire display (spectral density  ∝ *k*^−2^, where *k* is spatial frequency). The observer reported which stimulus component—*i.e*., which orientation (experiments of Figs. 3, 5, and S5) or image (experiment of Fig. 4)—was more visible by pressing one of two buttons on a joypad.

The experiment with simulated saccades (Fig. 3e, f) was identical, except that the fixation marker did not move, and participants maintained fixation at the center of the monitor throughout the trial. After a 450 ms delay to match the pre-saccadic exposure in the real-saccade experiment, the gratings moved on the monitor following the horizontal trace of a previously recorded saccade from the same observer, thereby replicating the retinal input signals delivered by a saccade while eliminating associated extraretinal influences. Examples of the recorded eye-position traces and analyses of fixation stability are reported in Supplementary Fig. S6.

### Data analysis

Data were analyzed using custom software written in MATLAB R2020a (MathWorks, Natick, MA). The recorded oculomotor traces were analyzed offline and segmented based on eye velocity. Eye movements with speeds exceeding 3°/s and amplitudes greater than $1{0}^{{\prime} }$ were classified as saccades. Trials were subjected to pre-established quality-control criteria, and only those with high-quality oculomotor measurements and accurate task execution were included in the analyses. Trials were excluded if participants blinked, made additional saccades besides the instructed one, or delayed saccade execution by more than 700 ms. Trials were also excluded if the gaze shift was incorrect, either because the landing error exceeded 1° or because saccade amplitude deviated from the requested gaze shift by more than 50%. On average across participants, 52% of trials were discarded (range: 22-66%). This selection ensured that the reported perceptual effects are based on trials with precise oculomotor execution and faithful reconstruction of the associated luminance transients.

For every observer, psychometric functions were estimated by fitting the data with cumulative Gaussian functions via a maximum-likelihood procedure^112^. The resulting psychometric functions were used to estimate the 50% performance threshold, the Probe contrast at which the Probe and Reference were equally visible (the Point of Subjective Equality, PSE). Statistical significance of difference in thresholds between psychometric functions was assessed for each participant using one-sided parametric bootstrap tests with 500 repetitions^113^. Sensitivity was defined as the inverse of the Probe contrast at the PSE. At the group level, statistical significance of sensitivity differences between saccade amplitudes was assessed with within-subject paired *t*-tests. Investigators were not blinded to experimental condition during outcome assessment, but exclusions and analyses followed predefined procedures.

To exclude confounds from possible differences in pre-saccadic exposure during the stimulus ramp, we also reanalyzed performance after equalizing the reaction times to small and large saccades (Fig. S3). For each participant in Fig. 3c, d, we discarded 1° saccade trials with long reaction times and 6° saccade trials with short reaction times so to match the mean pre-saccadic exposure duration across amplitudes. The remaining trials were then analyzed as described above.

### Power spectrum analysis and sensitivity prediction

Spectral analyses were conducted following the factorization approach described previously^36^. The key quantity is the power redistribution function *Q*(**k**, *ω*) resulting from eye movements—in this case, a saccade of a given amplitude. *Q* is a function of spatial frequency **k** and temporal frequency *ω*, and it specifies the temporal power distribution on the retina generated by the movement when viewing a stimulus of unit power at spatial frequency **k**.

Under the plausible assumption that the autocorrelation of the image on the retina and the second-order statistics of gaze displacement are statistically independent, the redistribution function *Q* enables estimation of the retinal input spectral density, *S*(**k**, *ω*), for arbitrary scenes:

1 $$\begin{array}{l}S({{{\bf{k}}}},\omega )=I({{{\bf{k}}}})Q({{{\bf{k}}}},\omega )\end{array}$$

where *I*(**k**) is the power spectrum of the external scene. By separating retinal image statistics and eye movements, this method enables spectral estimation with high temporal resolution at any desired spatial frequency **k**.

For every participant and saccade amplitude, *Q*(**k**, *ω*) was estimated from 100 recorded saccade traces. Each trajectory, ***ξ***(*t*), was isolated within a 512 ms segment centered on the movement. Displacements caused by pre- and post-saccadic drifts were removed, and the resulting traces were padded with stationary periods before and after the saccade. The padded traces were then used to construct the complex signal ${e}^{-2\pi j{{{{\bf{k}}}}}^{T}{{{\boldsymbol{\xi }}}}(t)}$, which describes how eye displacement modulates over time the retinal signal generated by a stimulus component at spatial frequency **k**. *Q*(**k**, *ω*) was estimated as the average periodogram of this signal:

2 $$Q\left({{{\mathbf{k}}}},\omega\right)=\left\langle \left|{\int}\,{{w}}(t)e^{-2\pi j{{{\mathbf{k}}}}^{T}{{{\boldsymbol{\xi}}}}\left(t\right)}e^{-2\pi j\omega t}\,dt\right|^{2}\right\rangle _{{{{\boldsymbol{\xi}}}}},$$

where 〈⋅〉_***ξ***_ denotes averaging across saccade traces and *w*(*t*) is a Hann raised cosine window used to reduce edge artifacts in the spectral estimate.

Padding the traces with stationary periods before and after the saccade is a standard procedure for reducing spectral leakage and, together with the Hann window, attenuated artifacts arising from the boundary discontinuities in the periodic repetition implicitly assumed by the Discrete Fourier Transform (DFT). This procedure preserved the frequency content of the luminance modulation delivered by the full saccade waveform and enabled functional interpretation of the resulting power spectra as characterizing the input signal that drives saccade-elicited neural responses. Note that, because of the properties of the DFT, restricting the spectral analysis to the isolated intra-saccadic segment would amount to estimating the spectrum of an artificial periodic waveform, thereby losing this functional interpretation: it would introduce spectral artifacts that obscure the actual luminance transition generated by the saccade (see Fig. S1e). Figure 1d and Fig. 4a show the average redistribution functions resulting from freely executed saccades of approximately 6°, 1°, and 0. 5° amplitude from a previously published free-viewing database (*N* = 9 observers)^36^, after averaging over orientation:

3 $$Q(k,\omega )= < Q({{{\bf{k}}}},\omega ){\ > \ }_{| {{{\bf{k}}}}|=k}.$$

To examine the effective strength of saccade-induced luminance modulations in driving visual responses, we filtered *Q*(*k*, *ω*) by the human temporal sensitivity function, *G*, and estimated the resulting total input power:

4 $$S(k)=\int_{0+}^{\infty }Q(k,\omega )| G(\omega,k){| }^{2}d\omega$$

where *G* was taken from classical psychophysical measurements obtained under retinal stabilization^43^, a condition that eliminates potential influences from eye movements:

5 $$\begin{array}{l}G(\omega ;k)=\left(6.1+7.3{\left\vert \log \left(\frac{\omega }{3}\right)\right\vert }^{3}\right)\omega k{e}^{-\frac{\omega+2k}{22.95}}\end{array}$$

Note that, unlike in [ref. ^36^], the filter in Eq. 4 is squared to estimate the output power. Figures 2c and 5b quantify the relative input power at the spatial frequencies of the Probe and the Reference as *S*(*k*_Probe_)/*S*(*k*_Ref_). Because our predictions were developed from transients generated by freely-executed (non-instructed) saccades, these figures also show that instructed saccades in our task produced power similar to that of amplitude-matched saccades recorded during free viewing of natural scenes. The curves in Figs. 1d, S1, and 5a were integrated using a space-time separable temporal sensitivity model^70^ to avoid the potential visual confound arising from the spatiotemporal coupling in Kelly’s filter.

To provide an intuition of the expected consequences of power redistributions from saccades, we modeled the average response of neuronal populations tuned to the orientation and frequencies of the Probe and the Reference:

6 $$\begin{array}{rcl}{\eta }_{Probe}&=&f({c}_{Probe}\sqrt{{S}_{A}({k}_{Probe})})+{{{\mathcal{N}}}}(0,\,{\sigma }_{noise})\\ {\eta }_{Ref}&=&f({c}_{Ref}\sqrt{{S}_{A}({k}_{Ref})})+{{{\mathcal{N}}}}(0,\,{\sigma }_{noise})\end{array}$$

where *c* is the contrast of the grating; *S*_*A*_(*k*) represents the strength of the visual input signals resulting from a saccade of amplitude *A* during viewing of a stimulus at spatial frequency *k*; *f* is a standard logistic function; and ${{{\mathcal{N}}}}$ Gaussian noise.

To account for the known nonlinear relation between input signals and neuronal responses in the primary visual cortex, we implemented a divisive normalization mechanism ^51^, which normalized the output of the model based on overall activity

7 $${\eta }_{i}=\frac{{\eta }_{i}}{{{{\Sigma }}}_{i}{\eta }_{i}+\gamma }$$

In each trial, the model compared the two responses and reported as most visible the grating that elicited the highest activity. The resulting psychometric functions evaluated over 30 contrast levels and 100 trials per level are reported in Fig. 2d.

### Reporting summary

Further information on research design is available in the Nature Portfolio Reporting Summary linked to this article.

## Supplementary information

Supplementary Information

Reporting Summary

Transparent Peer Review file

## Data Availability

The data generated in this study have been deposited in Code Ocean for public access (https://doi.org/10.24433/CO.9784697.v1).

## Code availability

The analysis code used in this study have been deposited in Code Ocean for public access (https://doi.org/10.24433/CO.9784697.v1).
