# Supplementary material for: What one sees depends on how far the eye has moved
Source: Nat Commun. 2026 Aug 30;17:10337. doi: 10.1038/s41467-026-76503-6 (PMC13623842; doi:10.1038/s41467-026-76503-6)
Supplement: Supplementary file 1 [file 41467_2026_76503_MOESM1_ESM.pdf]

# Supplementary Information

Y. H. Li, M. A. Cox, J. D. Victor, and M. Rucci

"What one sees depends on how far the eye has moved"

Nature Communications, 2026.

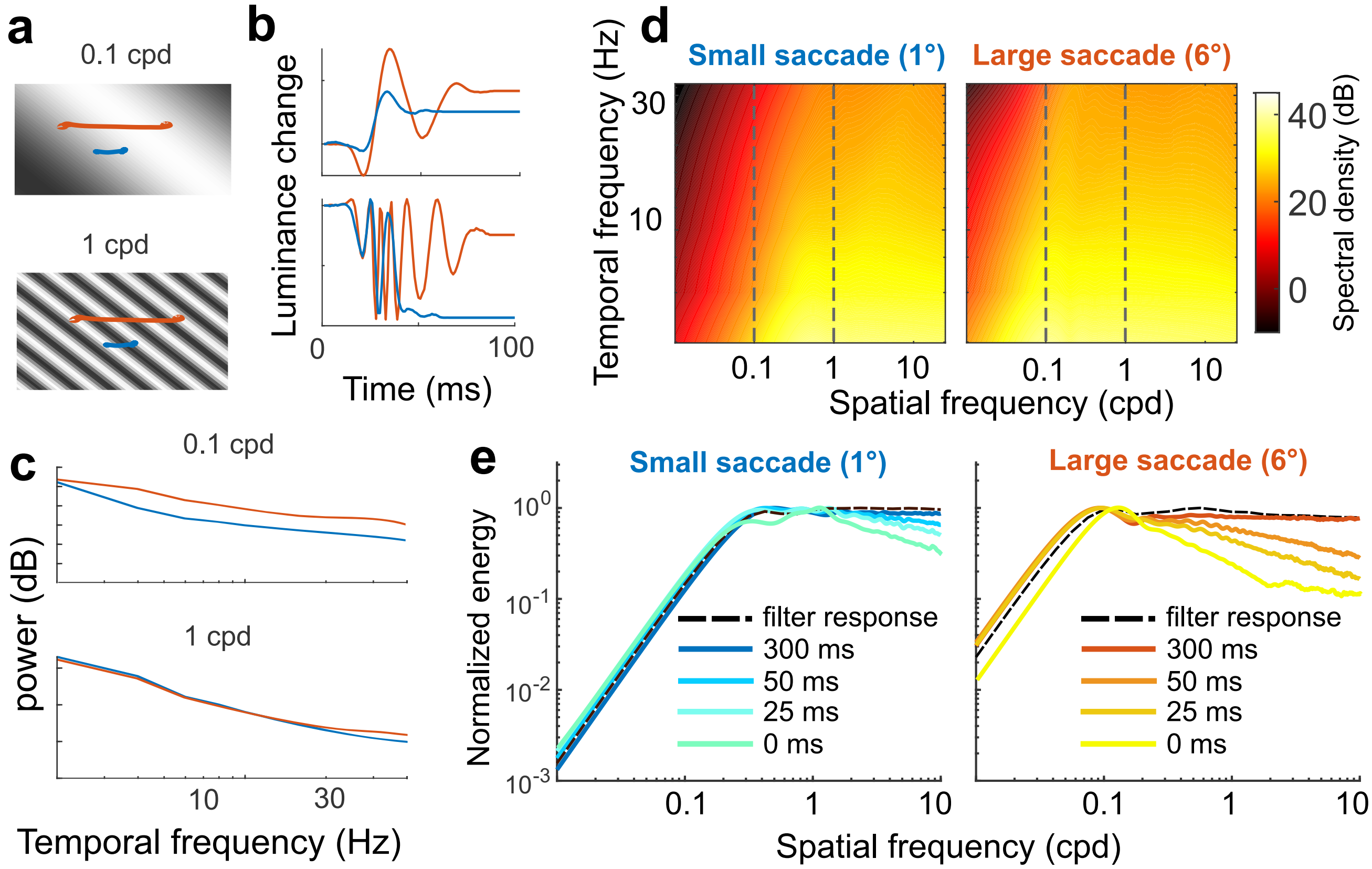


**Figure S1**: Visual input signals delivered by saccades of two amplitudes. **(a)** Example traces of a 6° (red) and a 1° (blue) saccade superimposed on gratings at low (top) and high (bottom) spatial frequencies. **(b)** Corresponding luminance modulations as a function of time. At low spatial frequencies, both saccades yield little luminance change (top). At sufficiently high spatial frequencies, once the saccades traverse more than one cycle of the stimulus, the amplitude of the modulation saturates. **(c)** Power spectra of the luminance transients in (b), averaged across many saccades and starting positions from $N$=9 observers. Note that the larger saccade yields stronger temporal modulations at low spatial frequencies. **(d)** Full spatiotemporal power spectra of the luminance transients resulting from 1° and 6° saccades (left and right panels, respectively), plotted as a function of stimulus spatial frequency (abscissa) and temporal frequency (ordinate). The two dashed lines correspond to the spectra shown in (c). In both panels, the heatmap value at each point $(k, \omega)$ represents the fraction of total power made available at temporal frequency $\omega$ by a saccade of the considered amplitude when viewing a grating at spatial frequency $k$. **(e)** Spectral estimates obtained with buffer periods of constant luminance of various durations, weighted by the temporal sensitivity of magnocellular ganglion cells[1]. For comparison, the black dashed line shows the ground truth, *i.e.*, the energy in the saccade-induced response of the same temporal filter, directly estimated via convolution with the luminance modulations. As buffer duration decreases, the spectral estimates progressively deviate from the filter response because of increased spectral leakage and artifacts arising from the boundary discontinuities introduced by truncation.

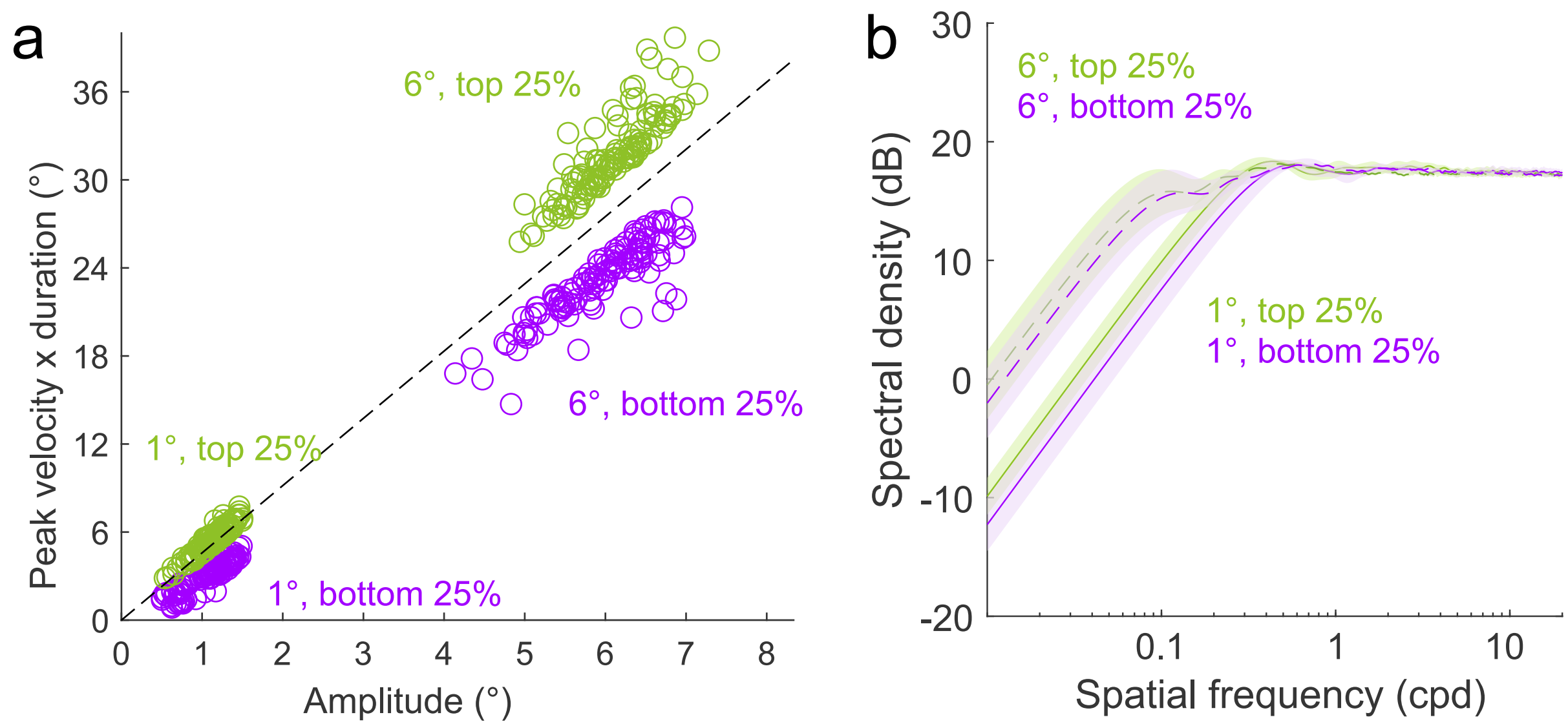


**Figure S2**: Predicted saccade influences are robust to trial-to-trial variability in saccade kinematics. **(a)** Saccades with the largest deviation from the main sequence for one representative observer. For each saccade performed in the experiment of Fig. 3, we computed the ratio $(v_{\text{peak}} \times T)/A$ (peak speed $v_{\text{peak}}$ times duration $T$, normalized by amplitude $A$) and examined its distribution separately for 1° and 6° saccades. Green and purple circles indicate, respectively, saccades in the first and fourth quartiles of either distribution. The dashed line shows the average main sequence, *i.e.*, the linear regression determined from all saccades. **(b)** Mean spectral density across participants for the four saccade groups. Shaded regions indicate ±1 SD. While faster and slower saccades at a fixed amplitude yield slightly different spectra, these differences are substantially smaller than the difference between saccade amplitudes. Data in both panels are from the $N$=9 participants in the experiment of Fig. 3c-d.

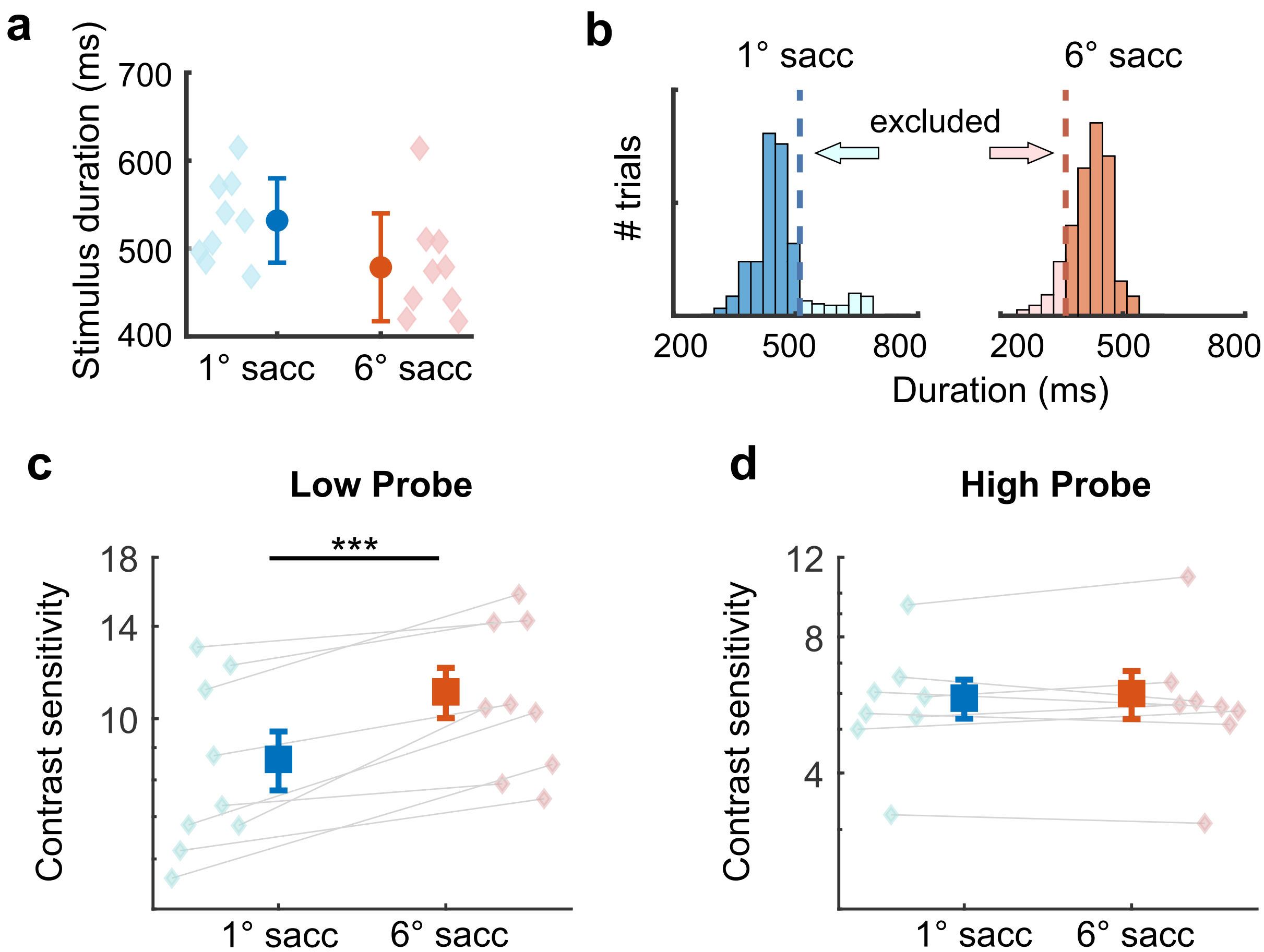


**Figure S3**: Control for pre-saccadic stimulus exposure. **(a)** Mean durations of the intervals of stimulus exposure before performing saccades of either 1° or 6° amplitude. On average, exposure was 53 ms longer with the smaller saccade. Diamonds mark the mean exposure duration for each individual; circles represent averages across participants. **(b)** Histograms of pre-saccadic stimulus exposure durations for a representative observer. The light-shaded regions mark the trials discarded to compensate for differences in saccade reaction times. **(c-d)** Sensitivity in the two conditions changes little after equalizing pre-saccadic exposure (***Low-Probe: $t(8) = 5.475$, $p < 0.001$, 95% CI = [1.39, 3.42], Cohen's $d = 0.83$; High-Probe: $t(7) = 0.561$, $p = 0.592$; two-sided paired $t$-tests).

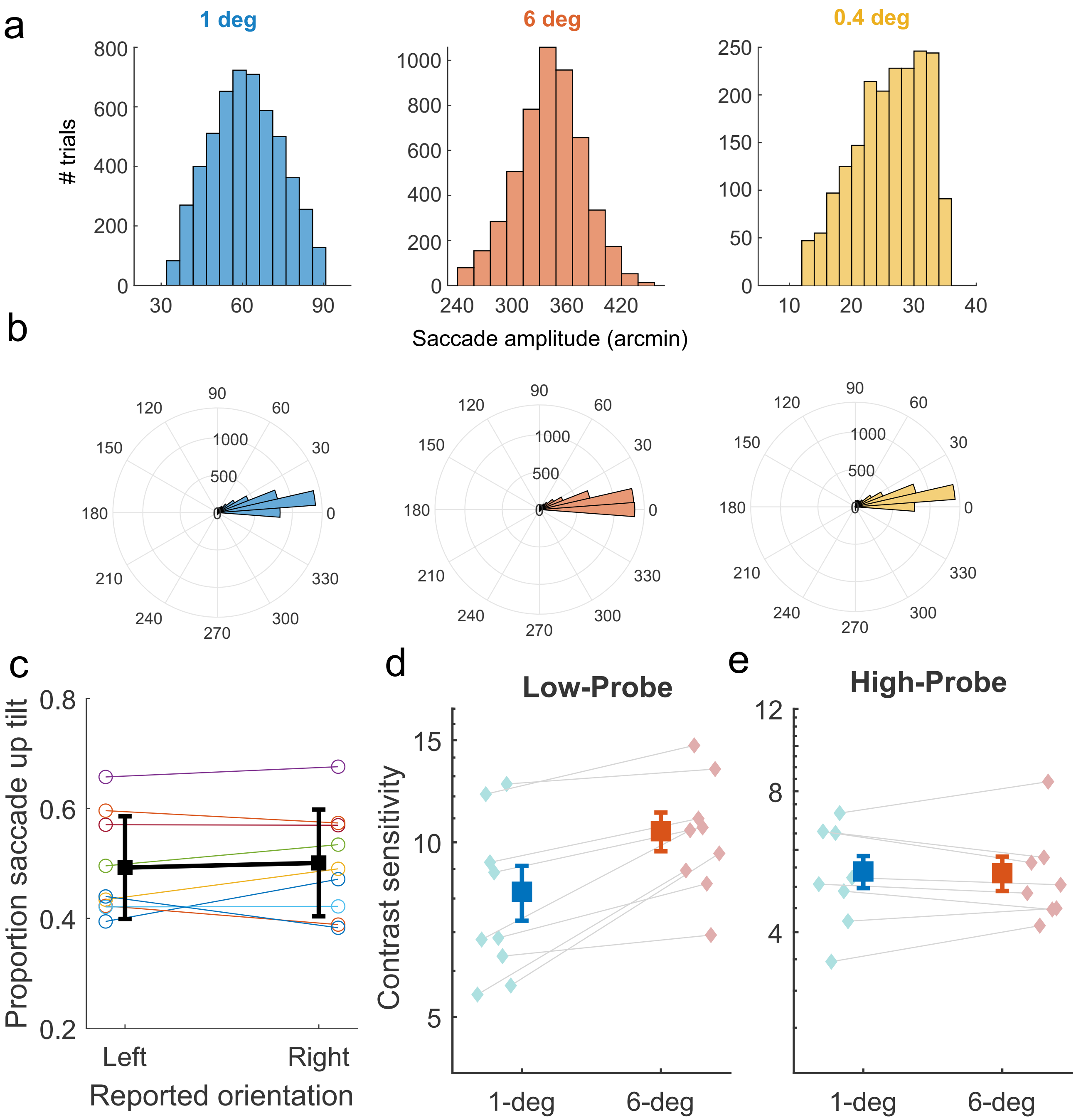


**Figure S4**: Saccade characteristics and consideration of possible orientation biases in the experiment of Fig. 3c-d. **(a–b)** Distributions of **(a)** saccade amplitudes and **(b)** saccade directions in the experiments of Figs. 3 and 5. **(c)** Proportion of saccades with an upward bias as a function of perceptual reports. Saccade orientation was not influenced by the perceptually more visible grating. **(d–e)** Perceptual results changed little when considering only saccades landing above or below the horizontal meridian so that the overall saccade direction was more parallel to the Probe. Graphic conventions are as in Fig. 3c ($N$=9 in $d$, and 8 in $e$).

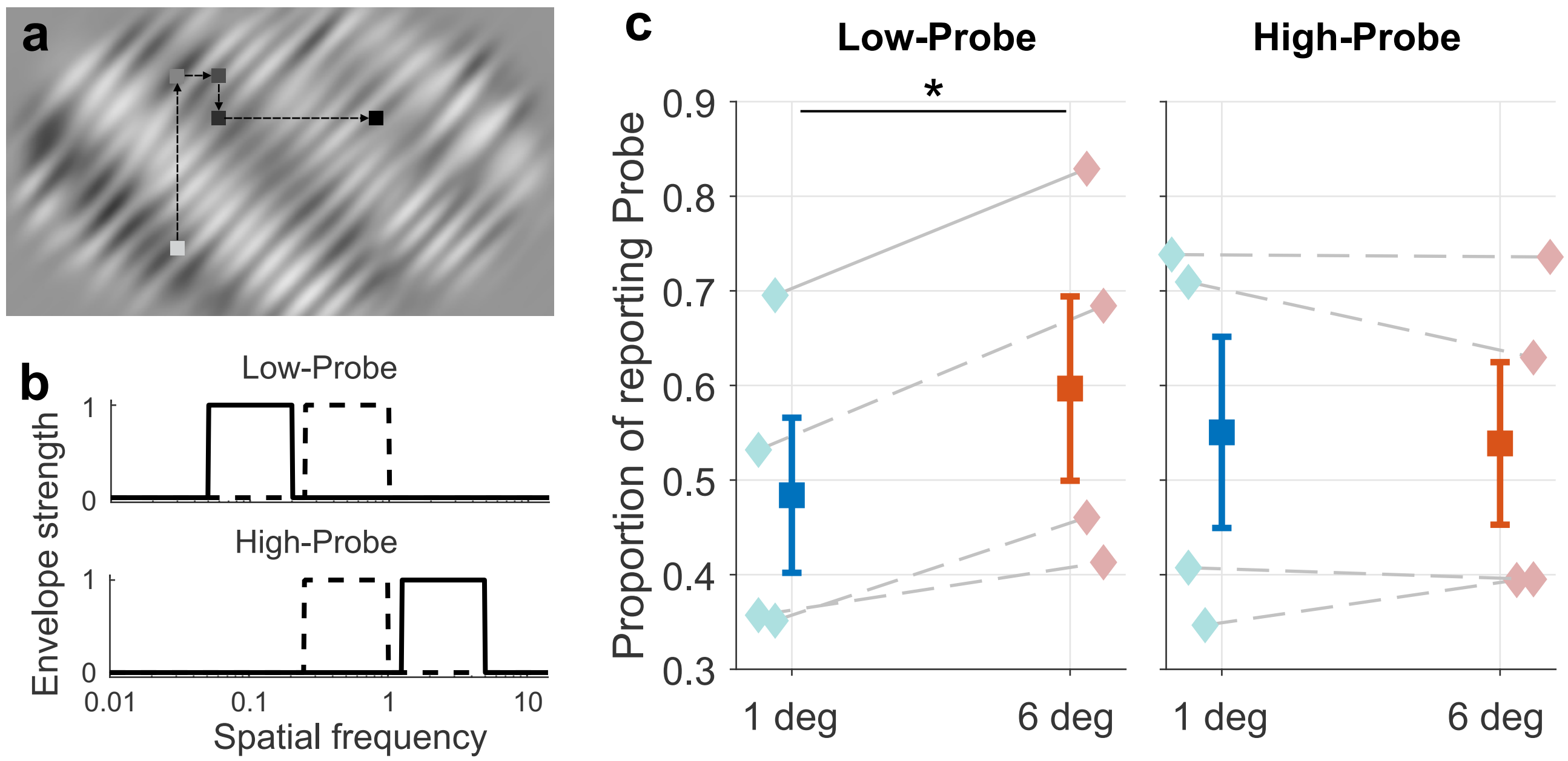


**Figure S5**: Visibility of broad-band stimuli following saccades alternating among different amplitudes and directions. **(a)** The experiment described in Fig. 3 was repeated with instructed saccades that varied randomly both in amplitude (1° or 6°) and in direction (up, down, left, or right). Each trial followed the same sequence of events described in Fig. 2a, with gaze position starting from the end of the last saccade. **(b)** Stimuli consisted of the superposition of two orthogonally oriented (±45°) band-pass white-noise patches (one-octave bandwidth) centered at 0.1 (Low-Probe), 0.5 (Reference), and 2.5 (High-Probe) cycles/deg. **(c)** Data from $N = 4$ observers in the two conditions in which the Probe was at either a lower or higher spatial frequency than the Reference. Graphic conventions are as in Fig. 3. Larger saccades improved Probe visibility in the Low-Probe condition (*$t(3) = 5.392$, $p = 0.012$, 95% CI = [0.05, 0.18], Cohen's $d = 0.63$; two-sided paired $t$-test), whereas performance was not measurably influenced by saccade amplitude in the High-Probe condition ($t(3) =$ -0.447, $p = 0.685$; two-sided paired $t$-test).

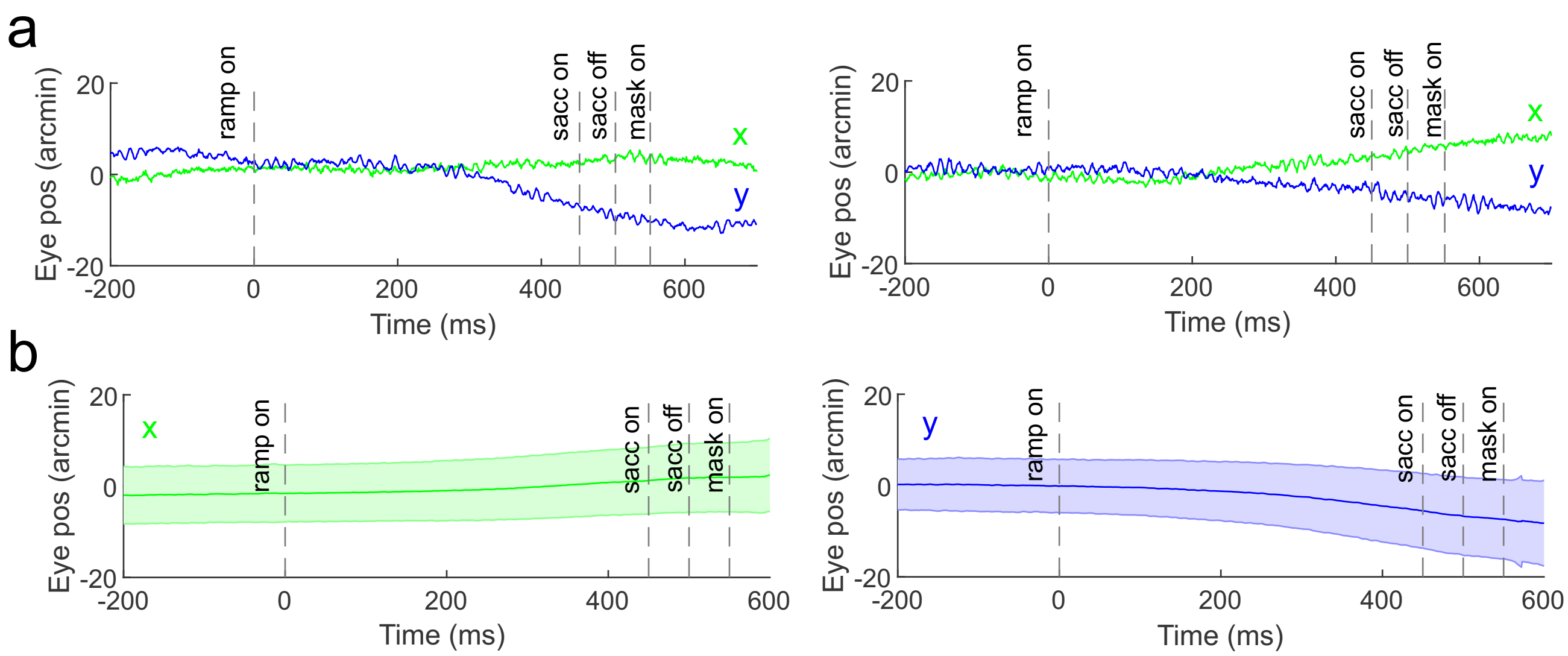


**Figure S6**: Gaze stability in the simulated saccade experiment of Fig. 3e–f. **(a)** Two examples of eye traces. Observers were instructed to maintain fixation for the entire duration of the trial. **(b)** Mean horizontal (left) and vertical (right) eye position across participants ($N$=4). Shaded error bars represent ±1 standard deviation. Fixation remained stable throughout the trial.

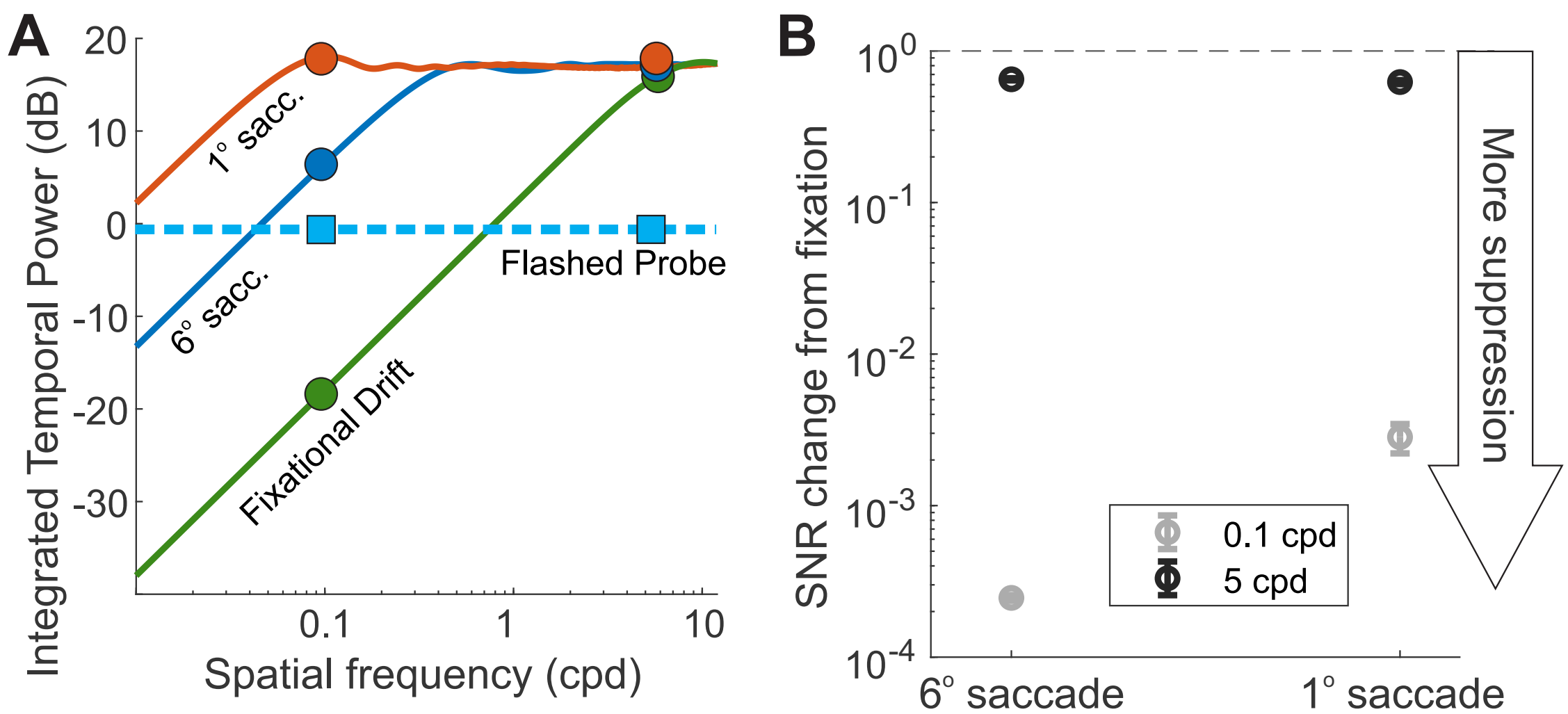


**Figure S7**: Strengths of luminance signals within the temporal bandwidth of human visual sensitivity[2] in typical experiments of saccadic suppression with visible backgrounds. **(A)** Temporal power delivered by fixational drifts ($P_{\text{Fix}}$), small and large saccades ($P_{\text{Sacc}}$), and a briefly flashed probe ($P_{\text{Probe}}$) as a function of the spatial frequency of the probe and the background. Squares mark the signal power of the probes at 0.1 and 5 cpd; circles mark the corresponding noise power—the power of the transients generated by the saccades and drifts on the background at those same spatial frequencies. Note that, because of the brief flash, $P_{\text{Probe}}$ remains constant across spatial frequencies, unlike the power resulting from eye movements. **(B)** Changes in Signal-to-Noise Ratios (SNRs) for intrasaccadic probes relative to when the same probes are flashed during fixation. At fixation, the SNR is defined as $P_{\text{Probe}}/P_{\text{Fix}}$. For an intrasaccadic probe, the SNR is $P_{\text{Probe}}/P_{\text{Sacc}}$. Consistent with classical results on saccadic suppression, the SNR decreases more for lower spatial frequency probes and larger saccade amplitudes. Data points represent averages ± one SD for the $N$=9 participants in the experiment of Fig. 3.

# References

1. Benardete, E. A. & Kaplan, E. The dynamics of primate M retinal ganglion cells. *Vis. Neurosci.* **16**, 355–368 (1999).

2. Watson, A. B. *et al.* Temporal sensitivity. *Handbook of perception and human performance* **1**, 6–1 (1986).
