# Supplementary material for: What one sees depends on how far the eye has moved
Source: Nat Commun. 2026 Aug 30;17:10337. doi: 10.1038/s41467-026-76503-6 (PMC13623842; doi:10.1038/s41467-026-76503-6)
Supplement: Supplementary file 3 [file 41467_2026_76503_MOESM3_ESM.pdf]

nature portfolio

Peer Review File

What one sees depends on how far the eye has moved

Corresponding Author: Dr Michele Rucci

This file contains all reviewer reports in order by version, followed by all author rebuttals in order by version.

Attachments originally included by the reviewers as part of their assessment can be found at the end of this file.

Version 0:

Reviewer comments:

Reviewer #1

(Remarks to the Author)
Summary

In their manuscript "What one sees depends on how far the eye has moved" the authors Li, Cox, Victor, and Rucci describe theoretical predictions and provide experimental support of the saccade amplitude-dependence of post-saccadic visual sensitivity. By spectral analysis of the visual changes induced by saccades, they show that saccades boost power in the low spatial-frequency (SF) domain and that increasing amplitudes shift the point where saturation in power occurs to lower SFs. Experimentally they further show that, when observers make saccades across superimposed orthogonal gratings – either low or high SF compared to a constant medium SF as reference – and report which one was more visible, larger saccades enhance contrast sensitivity to low SFs, but not high SFs. Finally, instructing even smaller saccades produced no reduction in sensitivity to low SFs, but increases in sensitivity to high SFs – a finding well predicted by the reference SF falling into the whitening region of the spectrum. Based on these results, the authors argue that saccades not only select spatial, but also spatial-frequency information.

Evaluation

As the main text is written in a clear and concise manner and figures present relevant information in a carefully structured and well-arranged fashion, the entire paper is really nice to read. The (relatively simple) experimental paradigm is methodologically sound and its results are straightforward. One has to appreciate the elegance by which theoretical predictions of the results are first carefully derived by modeling of saccade-induced luminance modulation and then confirmed by psychophysical measurements. The simulated-saccade experiment (even though only done with two observers and therefore unfortunately not really capable of providing a true comparison between saccade and fixation) further complements the already convincing picture of results. While I have no fundamental concerns regarding this paper – or rather, I like it a lot – I am not entirely convinced that it adds something critically new to the impressive list of simulations, findings, and proposals the authors have already put forward in the field. Theory has already been established by Mostofi et al. (2020), so this paper can be seen as providing experimental confirmation for their predictions by explicitly testing the effect of saccade amplitudes, complementing results by Boi et al. (2017). In this sense, as a follower of the authors' work over the years, none of the results or their implications presented here surprised me. As the authors note themselves, measured increases of contrast sensitivity fell short of theoretical predictions, and – what I see as the major shortcoming of the paper – many effects attributed to the saccade itself should actually be attributed to the combination of saccade and fixation periods before and after the saccade. Or rather, the respective contributions are by design impossible to separate. Therefore, some proposals outlined in the Discussion, such as those regarding saccadic suppression and spatial-frequency selection, seem logically inconsistent and beyond the explanatory power of the models and results and would strongly benefit from clarification and elaboration. Some more detailed (but unordered) concerns and suggestions are listed below.

Concerns and suggestions

- For both spectral analysis and their experimental paradigm, the authors use extended time windows that encompass pre- and post-saccadic stimulus presentations that by far outlast the duration of instructed saccades. While this is not per se a

problem, I doubt that it is correct to draw conclusions from this case to the intra-saccadic period, as it is done in the Discussion. For example, the authors conclude that “saccades actually yields weak signals at low spatial frequencies” (line 307), which greatly contradicts the fact that low SFs are within the ideal range of temporal sensitivity almost exclusively at saccadic speeds. Similarly, they report high power at high SFs (the saturation zone) even though such stimuli are routinely rendered invisible when presented during saccades (due to the high temporal frequencies, TFs, they induce; e.g., Castet, Jeanjean, & Masson, 2002). The only explanation I see why this pattern is reported is that it is not the intra-saccadic period the authors probe, but the trans-saccadic period, where visual processing and perception are not dominated by strictly saccadic transients, but by fixation, even though it seems the authors took great care in their spectral analysis to remove fixational eye movements. Figure 1d elucidates this well: If a strictly intra-saccadic period had been probed, then one would expect TF spectra to reveal a peak around the product of saccadic velocity and SF. They do not (and would anyway not be visible because the cut-off is set at 30 Hz), because the endpoints of the saccade introduce power in the low spatial frequencies. Thus, I would suggest to frame the Discussion more carefully, taking into account that the authors do not measure the power redistribution of the saccade per se, but of the change from pre- to post-saccadic fixation, within which the saccade’s visual consequences are embedded. Thus, I very much doubt that high-probe results would hold in a strictly saccadic setting, a limitation that should be made clear.

- The experiment uses orthogonal gratings, each 45 deg away from the instructed saccade direction, to ensure that luminance signals induced by them have comparable strength (that is, a parallel grating would not produce any luminance modulation). For that assumption to be valid, saccade vectors should be perfectly horizontal – a control analysis that could strengthen the validity of the manipulation.

- Why is it that spectral power in the saturation zone keeps on increasing – and by a decent amount of up to 10 dB in the 6-deg case? Similar analyses from previous papers have not shown this effect. If this is not an error, then the result should be explained and potential implications should be discussed.

- Although it should be clear to the attentive reader that it represents dB, Figure 1f could really use a legend label.

- I very much agree with the authors that saccades of increasing amplitudes enhance low SFs, reformatting spatial representations. Surely, this process is also needed, for instance to prevent fading of low SFs. Yet, I do not think the authors have provided sufficient evidence to claim that this is intended by the system, like spatial selection is intended. Up to this point, these perceptual changes are consequences of saccades – as they are consequences of simulated saccades, as the authors show – and could therefore be epiphenomena. The putative evidence that “saccade amplitude decreases when a high-pass filter is applied to the scene, which is consistent with the reduced low spatial frequency content” (l. 337) is presented in a way that neglects that these filters were applied centrally or peripherally, thus also changing observers’ spatial-selection strategies. Although I do not dislike the hypothesis per se, one could extend the argument: As saccades not only reformat power in the SF domain, but also in the orientation domain (leaving largely intact those parallel to the saccade direction, strictly during eye movements, of course; Schweitzer et al., 2025), should one argue that saccades are made in certain directions to briefly enhance the power in the respective bands in orientation space? Why would the system intend to do this and what would be the benefit of such a mechanism? To prevent the feeling of overstatement of results in this case (given the relatively small effects on contrast sensitivity), an extension or more careful formulation of this paragraph could help.

- “relative impact of the Probe and the Reference by means of their power ratio (the signal-to-noise ratio, SNR; Fig. 1g)” (lines 141-142) – is this truly the intended figure reference?

- I am curious about the quantitative mismatch between model predictions and experimental results, and their explanation. First, as the effect size in the low-probe condition is similar in real and simulated saccades, the authors suggest the “retinal component” of suppression. In other words, there seems to be a visual mechanism (specific to low SFs) that impairs contrast sensitivity and thereby counteracts the equally purely visual mechanism proposed in this paper that enhances contrast sensitivity. The trade-off between these two putative mechanisms would benefit from further elaboration: How would retinal image motion simultaneously enhance and suppress the same SF bands? Second, it remains unclear how the second explanation – that it is the system’s goal of “discounting variations in transient strength” – relates to the interpretation that saccades somewhat purposefully modulate these transients – it seems the opposite of what the authors want to convey with this paper.

- The argument that links the findings at hand to saccadic suppression and omission (starting at line 310) remains rather obscure. For one, how does the finding that saccadic suppression (that is, the reduction of contrast sensitivity to flashes around saccades) is strongest for low SFs relate to the finding at hand that saccades – especially the ones with large amplitudes that also produce stronger visual suppression – boost power in the low SF domain? To authors have found the contrary, that is, an enhancement of contrast sensitivity to low-SF stimulus and even argued previously that saccadic suppression might have dampened this perceptual effect. The “interference between transients” of probe and post-saccadic image seem to be the key, as well as the fact that the probe takes the form of a flash and thereby “preserves the spatial frequency distribution of the probe image” – but it remains entirely unclear how this mechanism works. While I am much in favor of the hypothesis that the visual contribution to the effect of saccadic suppression is significant, the authors should at least have to convincingly explain how their results can give rise to such a form of suppression, or at best show modeling or experimental evidence in favor of this view – especially because the current experiment at hand does not even test modulations introduced by strictly intra-saccadic probe images and can therefore not speak to this question.

(Remarks on code availability)

Reviewer #2

(Remarks to the Author)
This manuscript reports results from a study investigating the role of saccade amplitude on visual perception in human observers. The authors have asked participants to make fast, ballistic eye movements (saccades) of different amplitude. Initially, two amplitudes (1° vs. 6°) were tested. Remarkably, the smaller saccade reduced post-saccadic visual sensitivity specifically for low spatial frequency stimuli. Two further remarkable findings are reported. First, also simulated saccades led to this perceptual phenomenon. Second, reducing the saccade amplitude further (0.4°) revealed a theoretically predicted increase in sensitivity for high spatial frequency stimuli.
This study with its exciting results not only complements previous seminal studies from Rucci and colleagues. But its findings and theoretical implications will also trigger new work towards a better understanding of the interplay of eye movements and visual perception, in healthy participants, but also in various neuropsychiatric diseases.
As stated above, I consider this to be a very strong and exciting study, with state-of-the-art techniques (e.g. use of an dDPI, developed in this very lab), sound data analyses and rich implications for future studies. Accordingly, I have only a few suggestions to further improve the manuscript. I list my comments in chronological order:

Line 70 and Figure 1. The authors must better work out new results vs. a summary of existing literature. Figure 1 is extremely important for an understanding of the results, but to my understanding some of its content has been shown before in similar form, e.g. from Mostofi et al., Curr Biol, 2020?

Line 163, Figure: data from the representative participant should be indicated in Figures 2 c and 3 c-f.

Line 183: I am still surprised by the finding that also simulated saccades resulted in the selective influence of (simulated) saccade amplitude. The authors should further elaborate their discussion on this important point.

Line 311: somewhat related, the work by Diamond et al, J Neurosci, 2000 should be discussed in greater detail. These authors showed that saccadic suppression only occurs for real but not for simulated saccades. As a consequence, this work argues against the effect of masking.

Line 409: again, somewhat related. The authors should show eye traces for the fixation during simulated saccades. I wonder how stable fixation was during stimulus presentation and shortly after.

Line 418. The authors list a number of reasons why trials were discarded. This all seems plausible to me but in the end, the authors should indicate the proportion of trials per participant that was discarded.

Line 452. Was it 9 observers participating in the study (as indicated in line 353), or 18 observers, as stated here?

Minor:

Line 27: not all of our vision is about motion.

Line 106: arrow should read arrows

(Remarks on code availability)

Reviewer #3

(Remarks to the Author)
With their typical skill and insight, the authors report a subtle but statistically clear effect of gaze shifts on visual grating perception. The authors show that the spatiotemporal consequences of saccadic eye movements of different amplitudes (durations) on the visual image predict differential visibility of gratings with higher or lower spatial frequencies. The authors summarize the findings, “what one sees following a saccade critically depends on how far the eyes have moved”.

The findings are clear, and the manuscript is well constructed. However, the specificity of the findings and the particularity of their implications will restrict interest to rather narrow specialists.

MAJOR COMMENTS

(1) Although the perceptual effect is quantitatively clear and convincing insofar as it was tested, the practical consequences of the phenomenon are far from clear. Indeed, the length of the Discussion highlights the nuance of the observation. The perceptual reports are almost certainly overestimates based on the predictability of the reports derived from the repetition of particular gaze shifts and limitations of specific stimuli. The skeptic can wonder whether the effects will be as “considerable and highly consistent” (line 232) if saccades of unpredictable amplitude and direction are made or if mixtures of spatial

frequencies are judged. The highly-constrained and artificial testing conditions invite questions about the relevance for real-world perception. The discrepancy between the predicted 300% difference in perception and the observed 37% difference (line 264ff) reinforces this query. Also, if this manner of improving visibility mattered in natural vision, observers should control saccade amplitudes in order to improve visibility as predicted by the authors' framework. Do they? Also, in real-world terms, what objects or parts of objects have the spatial frequencies used for testing, and how often do humans make 0.4, 1.0, and 6.0 degree saccades in viewing them?

(2) The authors emphasize the image filtering in relation to saccade amplitude, but saccade velocity varies within amplitude. Indeed, in contrast to the assertion of the authors of "the tight relation between amplitude and speed" (line 94-95), other investigators have highlighted the variation in saccade velocity for a given amplitude. They describe variation of "saccade vigor" with task demands and other parameters. The authors' analysis should make specific predictions about perceptual variation across velocity for a given amplitude.

MINOR COMMENTS
The description of methods at lines 139-141 can be explained clearer for less sophisticated readers.

The description of methods at lines 146-147 is not as clear and explicit as could be. The free viewing condition is not included in the main methods.

The rationale for reporting the 0.4 degree saccade data later in the manuscript was not clear. The busy reader would appreciate more brevity. The skeptical reader would like to see how consistently participants produced 0.4 degree saccades.

(Remarks on code availability)

Version 1:

Reviewer comments:

Reviewer #1

(Remarks to the Author)
Evaluation
The authors Li, Cox, Victor, and Rucci have done a good job in revising their manuscript, but I still have two concerns, the first being the most relevant.

Concerns
1. Extended time windows around the saccade. In my previous review I voiced the concern that the authors' results – and that is in principle also the case for previous studies – are influenced by the pre- and post-saccadic intervals of stationarity that they add to the saccadic waveform. Their responses did not alleviate my concern, but created the impression that they did not know what I meant, which is why I would like to demonstrate it below using my own attempt to reproduce their results (given that this part of their code has not been made publicly available). Briefly, I simulated two compressed-exponential saccade trajectories according to the main sequence and computed the luminance changes induced when they are made across 100 bandpass-filtered noise images (SFs: 0.01, 0.5, 3 cpd), then computed the resulting TF spectra and weighted them by Kelly's (1979) sensitivity function, please see the figure I attached to this review. In a first condition (solid lines, circles) I wrapped the saccade waveform in either 250-ms noise-free fixation periods to replicate the authors' results: Indeed, larger saccades increase the resolvable power at low SFs, but not at high SFs, which is exactly what the authors report. In a second condition (dashed lines, triangles) I used only the saccade waveform, without fixation periods, to make the above point: While resolvable power is still boosted at low SFs, at higher SFs larger saccades decrease power – quite drastically – as higher saccadic velocities shift the spectrum towards even higher TFs, leaving increasingly little in the resolvable range. This is what Castet, Jeanjean & Masson (2002) found: Despite the distributing effect of the kinematic profiles of saccades and the broadband effect of the flash (both arguments used to respond to my concerns) the high-SF stimulus remained unresolvable. Another fitting example would be the indiscriminability of high-speed object motion – whether during saccades or fixation – if these objects are composed of high SFs (Schweitzer & Rolfs, 2020).
What restores power at high SFs, however, is a stable post-saccadic image, a highly efficient process known as post-saccadic masking (e.g., Duyck, Wexler, Castet, Collins, 2018; Schweitzer, Doering, Seel, Raisch, Rolfs, 2025). The authors wrote that "what temporal window is being analyzed ... does not confound saccades with fixations" and I still have to disagree. The current results stand because of the specific way spectral analysis and the experiment at hand was performed, that is, embedding the saccade in continuously present stimuli that enable masking of strictly saccade-induced transients. This is a limitation to the interpretation of the results that needs to be clarified. Even better, of course, would be a direct test to see whether the current results would hold when accounting for the pre- and post-saccadic period. That is, I have no doubt that the enhancement of low SFs at larger saccades holds but I would really like to see a version of their spectral analysis using strictly saccadic windows, believing that this adds value and novelty to the analyses presented already in previous publications.

2. The relationship to saccadic suppression. I believe that the authors have proposed an interesting mechanism relating their findings with classic findings of saccadic suppression. I believe it would be a good idea to include the additional figure in the

manuscript – it is not distracting as it makes a specific prediction about the relevance of the results at hand that may be critically evaluated in future experiments. I have two points to make though. First, Figure 1A is erroneous – as is Figure S1d (formerly Figure 1f) where the panels are now switched rendering the assignment large/small saccade incorrect – as there seems to have happened a switch-up between 1-deg and 6-deg saccades. Second, there is a logical inconsistency in the argument. The authors derive their predictions from a case where saccades spatiotemporally redistribute SF-power of natural scenes, whereas most saccadic-suppression experiments have taken great care to remove background structure by using a Ganzfeld-like setup. Notably, despite a great reduction in suppression strength, SF-selectivity persists even in near-total darkness (e.g., Burr, Holt, Johnstone, & Ross, 1982). How would these findings be accounted for in the proposed framework? Also, how is the given explanation compatible with "the absence of suppression observed for simulated—but not for real—saccades across uniform backgrounds or in complete darkness"? If anything, the mechanism the authors propose is visual-only and should be similar for real and simulated saccades - similar to what Idrees et al. (2020) found, but always requiring a structured background.

(Remarks on code availability)
The code for the model reported in this paper is available, but it does not generate figures, only a mat-file that comes without metadata. The code used for spectral analysis is unfortunately not available.

Reviewer #2

(Remarks to the Author)
The authors have convincingly addressed all my concerns. I have no further comments.

(Remarks on code availability)

Reviewer #3

(Remarks to the Author)
In the revised manuscript the authors have addresses very effectively the issues raised in review of the original submission. Textual and graphic clarification (e.g., Fig 1e) and experimental extension substantiate the phenomena and justify publication in a Nature journal.

A few minor comments:

(a) Discussing whether this effect on visibility can be incorporated into saccade planning, the authors include arguments derived from findings in mice. The logical relevance of these elements of the argument is unclear and seems to this reviewer even irrelevant.

(b) Figure 1c – The sequence of images in round apertures seem to relate to the image in 1a but not exactly. Seven images are (somehow) mapped onto 5 fixations. This potential confusion can be resolved by re-drawing or explaining.

(c) Figure 1d – The legend refers to "…± one standard deviation (shaded regions)", but such shaded regions are not obvious in the plot.

(d) Line 750 – "evect" should be "effect"

(Remarks on code availability)

Version 2:

Reviewer comments:

Reviewer #1

(Remarks to the Author)
I thank the authors for their efforts to clarify their choices, which have now become very clear. While I still disagree with some of their views (see minor comment below), I see no reason to further delay the publication of this substantial paper and congratulate the authors for their good work.

Minor comment: I am fully aware of the artifacts that arise when looking at isolated saccadic or saccade-like periods, and therefore also understand why the authors have deliberately created an experimental paradigm that deliberately avoided them by adding long stimulus ramps. However, only because spectral analysis may be unsuited to estimate very short, strictly saccadic transients does not mean that these considerations should be excluded in an a-priori manner. That is, the visual system shows very predictable responses to spectral splatter. For instance, temporal frequencies up to 200 Hz can be accurately discriminated when embedded in brief temporal windows, whereas prolonging those temporal windows logically lowers thresholds to more reasonable values around 50 Hz. Without a doubt, the reported results are valid in natural viewing

(which by definition always includes pre- and post-saccadic buffer periods) but high spectral power in high spatial frequencies during saccadic speeds may still seem highly unlikely for some workers in the field, if it were not for the introduced buffer periods. Just to mention one example, Gabor patches moving at saccadic speeds or beyond (even considering the spectral splatter that occurs as the stimulus rapidly enters and exits the receptive field) become invisible as a mere consequence of human temporal sensitivity (Rolfs, Schweitzer, Castet, Watson, & Ohl, 2025, Nat Comm). That is, even if pre- and post-saccadic endpoints were presented and saccadic velocity profiles were used. The same has been shown for motion smear, where the high spatial-frequency domain only regain relevance for the perceptual judgment as the duration of the post-saccadic window of stability is increased (Schweitzer, Doering, Seel, Raisch, & Rolfs, 2025, Psych Rev). In principle, these empirical results are now supported by the additional data points in Fig S1. Whether the authors want to explicitly mention them in their manuscript – in order to relate their results to a wider set of experimental settings or the critical choice of temporal windows – is of course up to them.

(Remarks on code availability)

Open Access This Peer Review File is licensed under a Creative Commons Attribution 4.0 International License, which permits use, sharing, adaptation, distribution and reproduction in any medium or format, as long as you give appropriate credit to the original author(s) and the source, provide a link to the Creative Commons license, and indicate if changes were made.
In cases where reviewers are anonymous, credit should be given to 'Anonymous Referee' and the source.
The images or other third party material in this Peer Review File are included in the article's Creative Commons license, unless indicated otherwise in a credit line to the material. If material is not included in the article's Creative Commons license and your intended use is not permitted by statutory regulation or exceeds the permitted use, you will need to obtain permission directly from the copyright holder.
To view a copy of this license, visit https://creativecommons.org/licenses/by/4.0/

Reply to Reviewer #1

Summary

In their manuscript "What one sees depends on how far the eye has moved" the authors Li, Cox, Victor, and Rucci describe theoretical predictions and provide experimental support of the saccade amplitude-dependence of post-saccadic visual sensitivity. By spectral analysis of the visual changes induced by saccades, they show that saccades boost power in the low spatial-frequency (SF) domain and that increasing amplitudes shift the point where saturation in power occurs to lower SFs. Experimentally they further show that, when observers make saccades across superimposed orthogonal gratings – either low or high SF compared to a constant medium SF as reference – and report which one was more visible, larger saccades enhance contrast sensitivity to low SFs, but not high SFs. Finally, instructing even smaller saccades produced no reduction in sensitivity to low SFs, but increases in sensitivity to high SFs – a finding well predicted by the reference SF falling into the whitening region of the spectrum. Based on these results, the authors argue that saccades not only select spatial, but also spatial-frequency information.

Evaluation

As the main text is written in a clear and concise manner and figures present relevant information in a carefully structured and well-arranged fashion, the entire paper is really nice to read. The (relatively simple) experimental paradigm is methodologically sound and its results are straightforward. One has to appreciate the elegance by which theoretical predictions of the results are first carefully derived by modeling of saccade-induced luminance modulation and then confirmed by psychophysical measurements. The simulated-saccade experiment (even though only done with two observers and therefore unfortunately not really capable of providing a true comparison between saccade and fixation) further complements the already convincing picture of results.

We thank the Reviewer for the kind comments and suggestions. We have added two additional subjects to the simulated-saccade experiment to further demonstrate that simple exposure to the visual transients is sufficient to yield the effects described in the study.

While I have no fundamental concerns regarding this paper – or rather, I like it a lot – I am not entirely convinced that it adds something critically new to the impressive list of simulations, findings, and proposals the authors have already put forward in the field. Theory has already been established by Mostofi et al. (2020), so this paper can be seen as providing experimental confirmation for their predictions by explicitly testing the effect of saccade amplitudes, complementing results by Boi et al. (2017). In this sense, as a follower of the authors' work over the years, none of the results or their implications presented here surprised me.

We are pleased that the Reviewer is familiar with our previous work. Indeed, the behavioral

observations in Boi et al. (2017) motivated the theoretical analysis in Mostofi et al. (2020), which led to the new, testable prediction that post-saccadic sensitivity should depend on the amplitude of the preceding saccade. The present study is the first to directly test and empirically establish this prediction in human observers.

While the logic leading to this prediction may not surprise a reader who has closely followed our earlier work, its experimental validation at the perceptual level is new and reveals a previously unrecognized functional role of saccades: they do not just select *where* information is sampled, but simultaneously filter *what* spatial information is encoded through their amplitude-dependent transients, thereby setting the scale of visual processing at the onset of each fixation.

This constitutes an important conceptual advance in our understanding of how perception unfolds during motor behavior. In response to the Reviewer's comment, we revised the Introduction and Discussion and redesigned Fig. 1 to clearly distinguish previously published theoretical background from the new empirical results and to articulate the novelty and broader significance of the work.

As the authors note themselves, measured increases of contrast sensitivity fell short of theoretical predictions and – what I see as the major shortcoming of the paper – many effects attributed to the saccade itself should actually be attributed to the combination of saccade and fixation periods before and after the saccade. Or rather, the respective contributions are by design impossible to separate.

This paragraph raises two distinct issues: (1) the apparent mismatch between theoretical predictions and measured changes in contrast sensitivity, and (2) the concern that the observed effects should be attributed to pre- and post-saccadic periods rather than to the saccade itself.

1. Relation between theory and experiment. We realize that our explanation of the quantitative comparison between theory and experiments in the previous version was not clear and rewrote this paragraph in the Discussion to avoid this misunderstanding. Psychophysical results do not "fall short" of the theoretical predictions. Our analysis in Fig. 2c concerns the input signals delivered by saccades to the retina, and the model in Fig. 2d was intended to quantify how a *parameter-free linear filter*, taken directly from the neurophysiological literature, would respond to this input—not to provide an exact quantitative prediction of perception. That a purely linear model would not quantitatively replicate perceptual performance is to be expected, given the well-established compressive nonlinearity between physical and perceived luminance and the known mechanisms for contrast gain control. To remove this source of confusion, we incorporated this nonlinearity into the model so that it can now be used to directly predict psychophysical performance. As shown in the revised Fig. 2d, this simple modification yields model responses that closely match the empirical data. We also emphasize that the measured effect is substantial: we observe a

∼40% improvement in sensitivity simply by changing how far the eyes move.

2. What is attributed to the saccade. The effects we describe are direct consequences of the saccade waveform; they are not driven by pre- and/or post-saccadic confounds. We address this point in detail below in this letter and in the revised Discussion, but it may be helpful to clarify upfront that our goal is to elucidate the perceptual consequences of the full spatiotemporal luminance flow generated by a saccade—a gaze shift that follows a specific kinematic profile. For this reason, we do not attempt to arbitrarily segment the movement into periods defined by instantaneous velocity. As explained below, this is a feature, not a limitation, of our approach: it is specifically designed to move beyond the common and intuitive, but oversimplified, view of saccades as uniform high-velocity translations.

Therefore, some proposals outlined in the Discussion, such as those regarding saccadic suppression and spatial-frequency selection, seem logically inconsistent and beyond the explanatory power of the models and results and would strongly benefit from clarification and elaboration. Some more detailed (but unordered) concerns and suggestions are listed below.

These comments, together with the elaborations in the Specific Comments below, indicate that our explanation of the generality of the approach was not sufficiently clear in the previous submission. The perceived "logical inconsistency" seems to originate from a lack of clarity regarding what our power analysis represents and how it captures spatiotemporal interactions.

Our framework is fundamentally general: the same spectral tools used in our study for stationary scenes can be applied directly to the spatiotemporal conditions of intra-saccadic experiments (*e.g.*, moving gratings or brief flashes), provided the specific modalities of stimulus presentation are taken into account. This allows for a conceptual interpretation of the retinal components of saccadic suppression based on the interaction of luminance transients, rather than purely on the spatial properties of the stimulus.

In light of these comments, and given that the implications for intra-saccadic perception are secondary to our main focus, we streamlined the Discussion. We condensed the section on saccadic suppression to avoid overextension and used the space to more clearly explain the underlying principles of our spectral analyses, their generality, and why their results may initially seem counterintuitive. We believe these revisions clarify the explanatory power of our approach without overextending its implications. Detailed changes are provided in our replies to the Specific Comments below.

Concerns and suggestions

- For both spectral analysis and their experimental paradigm, the authors use extended time windows that encompass pre- and post-saccadic stimulus presentations that by far outlast the duration of instructed saccades. While this is not per se a problem, I doubt that it is correct to draw conclusions from this case to the intra-saccadic period, as it is done in the

Discussion. For example, the authors conclude that "saccades actually yields weak signals at low spatial frequencies" (line 307), which greatly contradicts the fact that low SFs are within the ideal range of temporal sensitivity almost exclusively at saccadic speeds. Similarly, they report high power at high SFs (the saturation zone) even though such stimuli are routinely rendered invisible when presented during saccades (due to the high temporal frequencies, TFs, they induce; e.g., Castet, Jeanjean, & Masson, 2002). The only explanation I see why this pattern is reported is that it is not the intra-saccadic period the authors probe, but the trans-saccadic period, where visual processing and perception are not dominated by strictly saccadic transients, but by fixation, even though it seems the authors took great care in their spectral analysis to remove fixational eye movements.

We appreciate the Reviewer's comments, which helped us identify a number of concepts that needed clearer explanation in the manuscript. We understand that several of these points are already clear to the Reviewer, but we lay them down explicitly to avoid potential misunderstandings and revised the text to ensure that they will be clear to the reader.

1. What temporal window is being analyzed, and why this **does not** confound saccades with fixations.

Our spectral analyses quantify the luminance modulations generated by the *saccade waveform*, not by pre- or post-saccadic fixation or by other eye movements outside the saccade. We integrate power over the duration of the saccade because this provides a direct measure of the spatial information conveyed by the transient—*i.e.,* it gives a frequency representation of the signal that would effectively drive standard models of visual neurons immediately following a saccade. These results depend on the kinematics of saccades not on what happens before or after the saccade. We explicitly model the eye as stationary before and after the movement, thereby excluding fixational motion from the analysis. And results would be virtually identical if: (a) the analysis is conducted at finer time-frequency resolution via a spectrogram; or (b) if one assume the same speed profile to occur on a circular path so that the saccade end point coincides with the starting point. We clarified these points in the revised manuscript.

2. What was meant with "*saccades yield weak signals at low spatial frequencies.*"

This is a misunderstanding: the statement referred to the *relative* power generated by a saccade at low vs. high spatial frequencies: a low spatial frequency stimulus produces only small luminance changes as the eye translates, and in the extreme case of a uniform field, the saccade obviously generates no luminance modulation at all. The amplitude of the (radially-averaged) saccade-induced luminance modulation increases proportionally to the square of the spatial frequency until it saturates. Again, this power distribution does not depend on models or hypothetical assumptions: it is the actual input delivered by the saccade waveform without possible confounds from fixational eye movements.

3. Why the power generated from saccades appears to conflict with the standard intuition

that temporal frequency = instantaneous velocity $\times$ spatial frequency, $f = v\,k$?

The classical notion that the temporal frequency induced by eye movement can be approximated as peak instantaneous velocity $\times$ spatial frequency is useful as a first-order intuition. However, this relationship holds (a) *only for uniform motion*—a constant-velocity translation of infinite duration and (b) only for the frequency component orthogonal to the saccade motion. It does not provide an accurate description of the retinal input generated by brief movements like saccades, which follow highly stereotyped kinematic profiles with acceleration and deceleration phases. Because their velocity is not constant, these movements generate power across a broad range of temporal frequencies, as illustrated in Fig. 1d (now Fig. S1d). In other words, power is not concentrated at a single temporal frequency, but is spread across temporal frequencies as a direct consequence of the saccade waveform. It is also important to note that, because our goal is to understand how saccades redistribute the power of arbitrary visual scenes, our analysis follows the standard approach of averaging power spectra across all orientations at each spatial frequency, rather than considering only the special case of a grating orthogonal to the saccade direction.

4. Are our results informative for intra-saccadic visibility?

Our study focuses on what becomes visible following a saccade on a static scene, so this discussion point is not central and could be completely eliminated without affecting the significance of the article. However, we think that there is an interesting observation to be made: embracing the idea that the visual system exploits oculomotor-induced temporal transients implies a reconsideration of classical studies that flashed stimuli during saccades: these studies emphasized the action of the spatial content of the stimulus without considering the action of the flash itself.

The confusion seems to originate from the Reviewer directly applying the power redistribution caused by saccades during viewing static scenes (Fig. 1d) to moving stimuli (like in Castet et al) or probes briefly presented during saccades. Our approach can still be applied, but these stimuli yield different power distributions than the one shown in Fig. 1d, which need to be considered. The point made in the Discussion is that visibility of briefly flashed stimuli in classical experiments matches what one would expect based on the interaction between the transients generated by these stimuli and those resulting from eye movements on the background, when present. We provide a detailed description of this interaction in the reply to the last point in this letter, when the Reviewer asks again about saccadic suppression.

Figure 1d elucidates this well: If a strictly intra-saccadic period had been probed, then one would expect TF spectra to reveal a peak around the product of saccadic velocity and SF. They do not (and would anyway not be visible because the cut-off is set at 30 Hz), because the endpoints of the saccade introduce power in the low spatial frequencies.

Former Fig. 1d (now Supplementary Fig. S1d) quantifies how much power at each spatial

frequency a saccade makes available at each temporal frequency during viewing of a static scene. Because saccades have non-constant velocity profiles and finite duration, they distribute energy across a broad range of temporal frequencies. For this reason, these spectra correctly do not show power concentrated at a single temporal frequency.

In paradigms in which vision is probed with brief flashes during saccades, one should not expect a single dominant temporal frequency either: a brief flash inherently redistributes power broadly across temporal frequencies (the Fourier transform of an infinitesimally short pulse is flat). Thus, even in these conditions, the retinal input would not be restricted to a narrow temporal band.

Perhaps the Reviewer has in mind the situation of a grating at spatial frequency k translating at uniform velocity v (as in Castet et al., 2002). In that special case, the power of the external stimulus is concentrated at a single temporal frequency ($\omega = v\ k$). However, when such a stimulus is viewed during a saccade, the retinal spatiotemporal power is given by convolving this narrow spectral peak with the same broad redistribution function produced by the saccade (Fig. 1d). The resulting spectrum would indeed exhibit a peak at $\omega = vk$, but it would also show substantial spread across temporal frequencies because of the characteristics of saccadic motion. Within our framework, visibility would then depend on how much of this redistributed power falls within the temporal bandwidth of visual sensitivity. Consistent with empirical findings, a low-SF grating would yield much more effective power than a high-SF grating.

Thus, I would suggest to frame the Discussion more carefully, taking into account that the authors do not measure the power redistribution of the saccade per se, but of the change from pre- to post-saccadic fixation, within which the saccade's visual consequences are embedded. Thus, I very much doubt that high-probe results would hold in a strictly saccadic setting, a limitation that should be made clear.

We extensively revised the Discussion to better explain the significance of our findings, the approach used to develop our theoretical predictions, and the generality (and scope) of the framework (see pages 11-12). Following the Reviewer's suggestion, we also scaled back speculations on experimental paradigms that are not central to our study to ensure that the manuscript remains focused on our primary results. This round of reviews made clear that such discussion can be distracting without substantially more dedicated explanation.

As requested, we now explicitly clarify that (a) our predictions and empirical tests are based on the transients generated by saccades over stationary scenes, and (b) extending this framework to other paradigms—such as those involving briefly flashed stimuli—requires careful consideration of the additional transients produced by the stimulus presentation itself. For example, if stimuli are flashed, power is equally redistributed across a broad range of temporal frequencies for both the Probe and the Reference. Under these conditions, the relative influence of saccade-induced transients would be expected to differ from the effects

observed with stationary stimuli. The relevant text is on pages 14 and 15.

- The experiment uses orthogonal gratings, each 45 deg away from the instructed saccade direction, to ensure that luminance signals induced by them have comparable strength (that is, a parallel grating would not produce any luminance modulation). For that assumption to be valid, saccade vectors should be perfectly horizontal – a control analysis that could strengthen the validity of the manipulation.

This is an interesting observation, and we agree that it is important to exclude this possible confound. We added a supplementary figure (Fig. S4) to report the distribution of saccade directions. As shown by these data, saccades were predominantly horizontal, with small deviations from the instructed direction. These deviations had little consequences on the power delivered by the two components of the stimulus. To rule out possible influences, we examined whether saccade directions and perceptual reports interacted. We found no correlation between subjects' responses and saccade direction ($r < 0.01, p = 0.27$) . We also found no systematic bias in saccade direction and the orientation of the perceptually more visible grating. Furthermore, the perceptual enhancement of the larger saccade changes little if we only consider the saccades oriented more parallel to the probe.

In addition, we included the results of a new experiment (new Fig. 4) using hybrid images rather than oriented gratings. These images contain gratings in all orientations. The fact that the amplitude-dependent effect persists with these more naturalistic stimuli further demonstrates the robustness of the phenomenon and confirms that it does not rely on the precise orientation of gratings.

- Why is it that spectral power in the saturation zone keeps on increasing – and by a decent amount of up to 10 dB in the 6-deg case? Similar analyses from previous papers have not shown this effect. If this is not an error, then the result should be explained and potential implications should be discussed.

The apparent discrepancy between former Fig. 1g (now Fig. 1d) and earlier work (*e.g.*, Fig. 3 in Mostofi et al., 2020) is not an error; it arises because the two figures plot different quantities. Mostofi et al. reported the *total power* made available by the saccade within the temporal sensitivity bandwidth, whereas the present study plots the *effective input* to a linear perceptual model.

These two quantities differ in how power is weighted by the spatiotemporal sensitivity function $H(k, \omega)$ before integration across temporal frequencies. In Mostofi et al., the saccade-induced power $S(k, \omega)$ is directly weighted by H:

$$S_{\text{Mostofi}}(k) = \int S(k, \omega) \, H(k, \omega) \, d\omega.$$

In the present study, it is weighted by $|H|^2$, because we are interested in the *input that drives*

perceptual responses. In a linear system, the output power is proportional to the square of the transfer function:

$$S_{\text{here}}(k) = \int S(k,\omega)\,|H(k,\omega)|^2\,d\omega.$$

In our previous submission, as in Mostofi et al., the former Figure 1g used the sensitivity filter H provided by Kelly. This model varies with spatial frequency, and squaring the transfer function emphasizes the gain in the saturation region, leading to the observed increase. Thus, the increase observed in the previous version of Fig. 1d was a mathematical consequence of the filter's shape, not a difference in the saccades themselves.

To avoid confusion regarding these technical details, we updated Figs. 1d and 5a to use a space-time separable temporal sensitivity model (Watson, 1986). Unlike the Kelly model, the Watson filter's temporal profile does not vary with spatial frequency, which eliminates the increase in the saturation zone and aligns the visualization with previous work—as well as avoiding a potential confound due to spatiotemporal coupling in the filter itself. Crucially, the main effects and conclusions of our study remain robust regardless of whether the Kelly or Watson filter is employed. The relevant text is on page 22. We also added a note to the Methods section to alert the reader to the squaring of the filter in our analysis (line 526).

- Although it should be clear to the attentive reader that it represents dB, Figure 1f could really use a legend label.

This is now Supplementary Figure S1d. We added units to the colorbar.

- I very much agree with the authors that saccades of increasing amplitudes enhance low SFs, reformatting spatial representations. Surely, this process is also needed, for instance to prevent fading of low SFs. Yet, I do not think the authors have provided sufficient evidence to claim that this is intended by the system, like spatial selection is intended. Up to this point, these perceptual changes are consequences of saccades – as they are consequences of simulated saccades, as the authors show – and could therefore be epiphenomena. The putative evidence that "saccade amplitude decreases when a high-pass filter is applied to the scene, which is consistent with the reduced low spatial frequency content" (l. 337) is presented in a way that neglects that these filters were applied centrally or peripherally, thus also changing observers' spatial-selection strategies. Although I do not dislike the hypothesis per se, one could extend the argument: As saccades not only reformat power in the SF domain, but also in the orientation domain (leaving largely intact those parallel to the saccade direction, strictly during eye movements, of course; Schweitzer et al., 2025), should one argue that saccades are made in certain directions to briefly enhance the power in the respective bands in orientation space? Why would the system intend to do this and what would be the benefit of such a mechanism? To prevent the feeling of overstatement of results in this case (given the relatively small effects on contrast sensitivity), an extension or more careful formulation of this paragraph could help.

We fully agree with the Reviewer's distinction: our results demonstrate that saccade amplitude acts as a perceptual spatial filter, but they remain agnostic as to whether this filtering function is an "intended" goal of saccade planning beyond foveation. The spatiotemporal reformatting occurs as a direct physical consequence of the eye movement. This raises the hypothesis that saccade planning may also include consideration of this perceptual outcome, but it was not our intention to imply that our study demonstrates such intent. We rewrote the relevant paragraph in the Discussion to ensure that it is clear that this is a hypothesis, not a conclusion (second paragraph of page 15).

Regarding the comment on Cajar et al. (2016), the Reviewer makes a valid point that applying filters centrally versus peripherally could, in principle, alter spatial-selection strategies. However, Cajar et al. report that, relative to low-pass filtering, high-pass filtering reduces saccade amplitude across all filtering configurations—central, peripheral, and whole-image (see their Fig. 2b). We revised the text to clarify this, and we now cite several other studies that reported similar results. These modulations in saccade amplitude are qualitatively consistent with the use of saccades as spatial filters, even though dedicated experiments will be needed to test this hypothesis.

We thank the Reviewer for bringing the relevant article by Schweitzer et al. (2025) to our attention. We now cite it in the revised manuscript.

- "relative impact of the Probe and the Reference by means of their power ratio (the signal-to-noise ratio, SNR; Fig. 1g)" (lines 141-142) – is this truly the intended figure reference?

This figure reference was not correct. We now updated it to refer to the appropriate panel (Fig. 2c).

- I am curious about the quantitative mismatch between model predictions and experimental results, and their explanation. First, as the effect size in the low-probe condition is similar in real and simulated saccades, the authors suggest the "retinal component" of suppression. In other words, there seems to be a visual mechanism (specific to low SFs) that impairs contrast sensitivity and thereby counteracts the equally purely visual mechanism proposed in this paper that enhances contrast sensitivity. The trade-off between these two putative mechanisms would benefit from further elaboration: How would retinal image motion simultaneously enhance and suppress the same SF bands? Second, it remains unclear how the second explanation – that it is the system's goal of "discounting variations in transient strength" – relates to the interpretation that saccades somewhat purposefully modulate these transients – it seems the opposite of what the authors want to convey with this paper.

As mentioned in our reply to the general comments above, this concern stems from a paragraph in the Discussion that was not sufficiently clear. In the previous version, we referred to the integrated temporal power on the retina as a "perceptual prediction," which was misleading. Our goal was to illustrate the qualitative consequences of the input signals (*i.e.*, how changes in saccade kinematics redistribute stimulus power within the temporal bandwidth

of sensitivity), not to provide a quantitative prediction of performance.

It is well-established that both perceived contrast and luminance do not linearly follow the input to the retina. The original paragraph was intended to summarize several nonlinear mechanisms reported in the literature that mediate this relationship. However, our discussion of their hypothesized functions contributed to the confusion. These nonlinear mechanisms—such as Fechner or Stevens-type psychometric functions—attenuate, but do not eliminate, changes in perceptual responses as a function of the strength of input signals.

To remove this source of confusion, we now incorporated a standard compressive nonlinearity between physical and perceived contrast into the model. With this addition, the model closely matches the empirical effect size, predicting a substantial ∼40% improvement in sensitivity as saccade amplitude increases. We rewrote the relevant paragraph in the Discussion to clarify these points and eliminate potential misunderstandings. The new text is in the second paragraph of page 12.

We address the Reviewer's specific comments regarding the retinal component of saccadic suppression in our response to the following point.

- The argument that links the findings at hand to saccadic suppression and omission (starting at line 310) remains rather obscure. For one, how does the finding that saccadic suppression (that is, the reduction of contrast sensitivity to flashes around saccades) is strongest for low SFs relate to the finding at hand that saccades – especially the ones with large amplitudes that also produce stronger visual suppression – boost power in the low SF domain? To authors have found the contrary, that is, an enhancement of contrast sensitivity to low-SF stimulus and even argued previously that saccadic suppression might have dampened this perceptual effect. The "interference between transients" of probe and post-saccadic image seem to be the key, as well as the fact that the probe takes the form of a flash and thereby "preserves the spatial frequency distribution of the probe image" – but it remains entirely unclear how this mechanism works.

This comment concerns a paragraph in the Discussion in which we speculated on how our findings may relate to classical results on saccadic suppression. Within our framework, suppression of intra-saccadic probes presented against a visible background can be naturally interpreted as arising from interactions between the saccade-induced background transients—the focus of the present study—and the luminance transients generated by the probe itself.

Our results indicate that visibility depends on the spatial information conveyed by luminance transients within the temporal bandwidth of visual sensitivity. In saccadic-suppression paradigms that are not conducted in complete darkness, two distinct sources of transients coexist: the transient produced by the probe flash, which acts as the *signal*, and the transient generated by eye movements on the background—either by the saccade or, when the probe is presented at fixation, by ocular drift—which acts as masking *noise* for probe detection.

Crucially, these two components scale differently with spatial frequency (see Fig. 1 below). The power of the probe transient does not depend on spatial frequency, because a brief flash redistributes power broadly across temporal frequencies regardless of the probe's spatial content. In contrast, the power of the background transient generated by either saccades or drifts depends strongly on both the spatial frequency of the background and the type of eye movement. Saccades and ocular drifts yield comparable power at high spatial frequencies. Consequently, the signal-to-noise ratio (SNR) for a high–spatial-frequency probe is expected to be similar whether the probe is presented during a saccade or during fixation. In contrast, for a low–spatial-frequency probe, the SNR is much higher at fixation than during a saccade, because ocular drift generates little temporal power at low spatial frequencies. The larger the saccade, the stronger the transient from the background, and the lower will be the SNR. This pattern is consistent with the classical findings that saccadic suppression is strongest at low spatial frequencies (*e.g.*, Burr et al, Nature 1994) and scales with saccade amplitude (*e.g.*, Ridder & Tomlinson, Vis Res 1997).

In sum, there is no contradiction between suppression of flashed probes during saccades and the post-saccadic enhancement we report: the increase in background power at low spatial frequencies provides more effective drive for stationary scenes (enhancement), but at the same time may act as masking noise for detecting brief flashes (suppression). We revised the Discussion to make this correspondence clear (see second paragraph of page 14 and first paragraph of page 15).

While I am much in favor of the hypothesis that the visual contribution to the effect of saccadic suppression is significant, the authors should at least have to convincingly explain how their results can give rise to such a form of suppression, or at best show modeling or experimental evidence in favor of this view – especially because the current experiment at hand does not even test modulations introduced by strictly intra-saccadic probe images and can therefore not speak to this question.

We rewrote the relevant paragraph in the Discussion to provide a clearer intuition of how the saccadic enhancements we report could, in principle, also contribute to the effects observed with intrasaccadic probes. To address the Reviewer's request for more concrete evidence, we provide a quantitative analysis in Figure 1 of this letter showing how the SNR of a flashed probe is selectively reduced at low spatial frequencies by the very transients we describe in our study.[1]

We believe this provides a parsimonious visual mechanism that aligns our results with classical findings of saccadic suppression. We regard this as an interesting hypothesis emerging from our study. However, we are reluctant to expand its discussion much further in the manuscript to avoid distracting from our main focus on post-saccadic visibility. If the revised concise treatment remains insufficient, the discussion of intrasaccadic visibility can also

[1] To maintain the focus of the study on post-saccadic enhancements, we have not included this analysis in the manuscript, but we would be glad to incorporate it should the Reviewer and Editor find it helpful.

be removed entirely, as it does not affect the primary conclusions or the significance of our work.

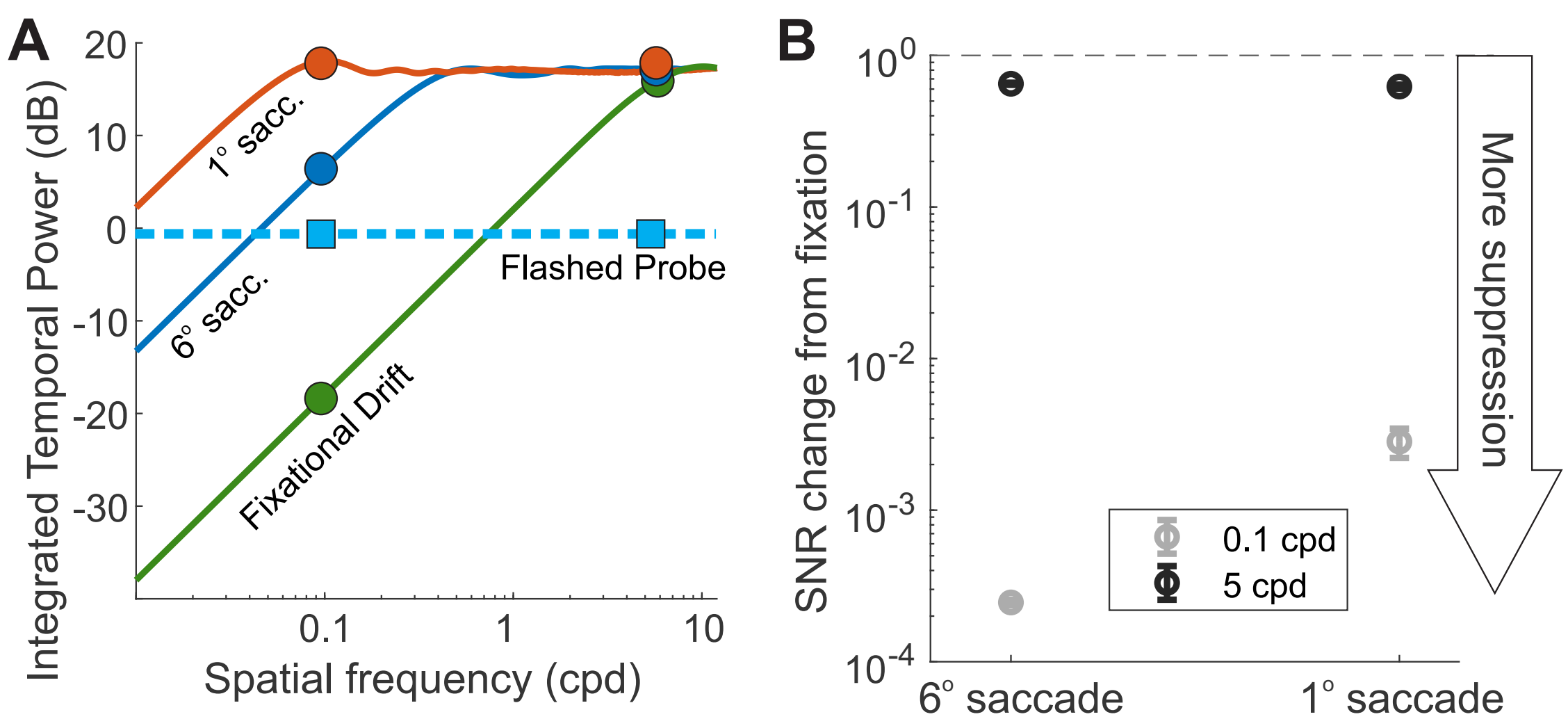

Figure 1: Strengths of visual signals within the temporal sensitivity bandwidth in typical experiments of saccadic suppression with visible backgrounds. **(A)** Temporal power delivered by fixational drifts (P_{Fix}), small and large saccades (P_{Sacc}), and a briefly flashed probe (P_{Probe}) as a function of the spatial frequency of the probe and the background. Squares mark the signal power of the probes at 0.1 and 5 cpd; circles mark the corresponding noise power—the power of the transients generated by the saccades and drifts on the background at those same spatial frequencies. Note that, because of the brief flash, P_{Probe} remains constant across spatial frequencies, unlike the power resulting from eye movements. **(B)** Changes in Signal-to-Noise Ratios (SNRs) for intrasaccadic probes relative to when the same probes are flashed during fixation. At fixation, the SNR is defined as $P_{\text{Probe}}/P_{\text{Fix}}$. For an intrasaccadic probe, the SNR is $P_{\text{Probe}}/P_{\text{Sacc}}$. Consistent with classical results on saccadic suppression, the SNR decreases more for lower spatial frequency probes and larger saccade amplitudes. Data points represent averages ± one SD for the N=9 subjects in the experiment of Fig. 3.

Reply to Reviewer #2

This manuscript reports results from a study investigating the role of saccade amplitude on visual perception in human observers. The authors have asked participants to make fast, ballistic eye movements (saccades) of different amplitude. Initially, two amplitudes (1° vs. 6°) were tested. Remarkably, the smaller saccade reduced post-saccadic visual sensitivity specifically for low spatial frequency stimuli. Two further remarkable findings are reported. First, also simulated saccades led to this perceptual phenomenon. Second, reducing the saccade amplitude further (0.4°) revealed a theoretically predicted increase in sensitivity for high spatial frequency stimuli. This study with its exciting results not only complements previous seminal studies from Rucci and colleagues. But its findings and theoretical implications will also trigger new work towards a better understanding of the interplay of eye movements and visual perception, in healthy participants, but also in various neuropsychiatric diseases. As stated above, I consider this to be a very strong and exciting study, with state-of-the-art techniques (e.g. use of an dDPI, developed in this very lab), sound data analyses and rich implications for future studies. Accordingly, I have only a few suggestions to further improve the manuscript. I list my comments in chronological order:

We thank the Reviewer for the positive evaluation of our work and for the careful reading of the manuscript. All of the Reviewer's suggestions have been incorporated into the revised version, as detailed below. For clarity, in this letter, the Reviewer's comments are reported in blue and our responses in black.

Line 70 and Figure 1. The authors must better work out new results vs. a summary of existing literature. Figure 1 is extremely important for an understanding of the results, but to my understanding some of its content has been shown before in similar form, e.g. from Mostofi et al., Curr Biol, 2020?

We agree that the previous version of the manuscript did not clearly distinguish between material derived from earlier work and the new contributions of the present study. All panels in Figure 1 are based on analyses of the type introduced in Mostofi et al. (2020). Here, they are re-presented and adapted specifically to motivate the central hypothesis tested in this paper and to provide the theoretical background needed to interpret the new experiments, particularly for readers not familiar with our earlier work. The new empirical and modeling results begin with Figure 2.

In response to the Reviewer's comment, we revised the first three paragraphs of the Results to make clear that Figure 1 summarizes previously published theoretical analyses and to better convey the perceptual predictions tested in this study. We also modified Figure 1 to emphasize its conceptual role and provide a more intuitive understanding of the predictions, while moving more technical details to a new Supplementary Figure 1.

Line 163, Figure: data from the representative participant should be indicated in Figures 2

c and 3 c-f.

We updated both figures to clearly mark the data from the representative participant shown in Fig. 3a–b. Data points from this individual are now indicated by symbols with thicker, darker borders in both Figs. 2 and 3. These changes allow the reader to track the representative observer's performance across different conditions.

Line 183: I am still surprised by the finding that also simulated saccades resulted in the selective influence of (simulated) saccade amplitude. The authors should further elaborate their discussion on this important point.

We agree that the simulated-saccade results are important, as they provide a direct test of the retinal contribution to post-saccadic visibility. Following the Reviewer's comment, we increased the sample size for this experiment by adding two additional participants, which further confirms the robustness of the effect.

We also expanded the Discussion to elaborate on the significance of this finding (lines 294–298). By maintaining fixation while viewing spatiotemporal signals that mimic those generated by saccades, observers experience a redistribution of stimulus power across temporal frequencies similar to that produced by real eye movements. The fact that these simulated transients reproduce the selective influence of saccade amplitude indicates that the effect is driven by the spatial information conveyed by luminance transients, rather than by extra-retinal signals.

Line 311: somewhat related, the work by Diamond et al, J Neurosci, 2000 should be discussed in greater detail. These authors showed that saccadic suppression only occurs for real but not for simulated saccades. As a consequence, this work argues against the effect of masking.

We expanded the Discussion to clarify how our hypothesis builds upon the important work by Diamond et al. (2000). Our proposal is fully consistent with their findings, as the revised text now makes explicit.

Diamond et al. showed strong suppression for real saccades but little or no suppression for simulated saccades under conditions in which retinal stimulation from the display is minimal (e.g., uniform backgrounds or darkness), supporting an essential extra-retinal contribution. Our discussion concerns a complementary regime: paradigms in which probes are presented on a visible, structured background, where saccades generate substantial luminance transients in the background. In that case, these background transients can act as masking noise relative to the probe-flash transient, providing a plausible *retinal* contribution that would be minimized in uniform-field or dark conditions.

To avoid any ambiguity, we revised the Discussion to clearly delineate these stimulus-dependent regimes and to better explain how saccade-induced transients could contribute to the retinal component of saccadic suppression, alongside the extra-retinal mechanisms emphasized by Diamond et al. The relevant text is on pages 14-15.

Line 409: again, somewhat related. The authors should show eye traces for the fixation during simulated saccades. I wonder how stable fixation was during stimulus presentation and shortly after.

We added Supplementary Figure S6, which displays representative eye traces from a single participant (top row) and the average eye position across all subjects (bottom row). These data demonstrate that participants were able to maintain accurate fixation throughout the trial.

Line 418. The authors list a number of reasons why trials were discarded. This all seems plausible to me but in the end, the authors should indicate the proportion of trials per participant that was discarded.

We updated the Methods section (Lines 481–484) to explicitly report the proportion of discarded trials. To ensure the highest data quality and eliminate potential artifacts from inaccurate saccades or poor fixation, we applied stringent exclusion criteria. After applying these filters, we retained an average of 52% of the trials across all participants.

Line 452. Was it 9 observers participating in the study (as indicated in line 353), or 18 observers, as stated here?

We corrected this inconsistency and clarified the sample sizes throughout the revised manuscript. With the new data added in this revision, 12 observers participated in the experiments reported in this study. The number 9 refers instead to the separate free-viewing database used only for the power analyses of natural saccades (bold symbols in Figs. 2c and 5b, and the corresponding power distributions in Figs. 1d and 5a), in which 9 observers freely viewed natural scenes (from Mostofi et al., 2020). We revised the text here and in the Subjects section of the Methods to make this distinction explicit and ensured that the relevant N is stated in each figure caption.

Minor:

Line 27: not all of our vision is about motion.

We agree, and our study in fact provides an example of how temporal changes in the retinal input can be used to encode information about static scenes. We modified this introductory paragraph to more clearly convey the intended meaning that changes in input signals provide useful information not only about object motion, but also about the spatial structure of stationary objects.

Line 106: arrow should read arrows

We corrected the typo.

Reply to Reviewer #3

With their typical skill and insight, the authors report a subtle but statistically clear effect of gaze shifts on visual grating perception. The authors show that the spatiotemporal consequences of saccadic eye movements of different amplitudes (durations) on the visual image predict differential visibility of gratings with higher or lower spatial frequencies. The authors summarize the findings, "what one sees following a saccade critically depends on how far the eyes have moved".

The findings are clear, and the manuscript is well constructed. However, the specificity of the findings and the particularity of their implications will restrict interest to rather narrow specialists.

We thank the Reviewer for the critical evaluation of our manuscript. The comments helped us strengthen both the presentation of our conclusions and the articulation of the significance of the work.

If we understand the concern, its essence is that the effects we report may rely on tightly controlled laboratory conditions and might not play a significant role during natural viewing. To directly address this issue, the revised manuscript includes new experiments and control analyses showing that the effect: (a) persists despite trial-to-trial variability in saccade kinematics: (b) generalizes to more naturalistic stimuli and (c) to conditions in which saccade direction and amplitude vary unpredictably; and (d) is present even when similar spatiotemporal transients are passively experienced during fixation. We also revised the text to clarify that the mechanism we describe is general—saccade-induced transients vary systematically with amplitude—and that its perceptual consequences are substantial: changes in amplitude of only a few degrees produce changes in contrast sensitivity on the order of ∼40%!

In light of these results, we respectfully disagree that the implications of our work are of interest only to a narrow group of specialists. Saccades are among the most frequent motor actions humans perform, and it is widely assumed that their function is limited to the obvious one: foveation. Here, we show that this is not the case: in addition to selecting *where* information is sampled, they also shape *what* information is encoded—*i.e.*, the spatial scale of the information encoded at the onset of each fixation—through their amplitude-dependent transients. This phenomenon has direct relevance for multiple domains, including basic visual neuroscience, perceptual psychology, oculomotor research, and clinical contexts in which saccade amplitude is altered (*e.g.*, aging, neurological disease, or pathological eye movements). We extensively revised the manuscript, including substantial rewriting of the Discussion, to more clearly explain the novelty, generality, and broader significance of the findings.

Major comments

(1) Although the perceptual effect is quantitatively clear and convincing insofar as it was

tested, the practical consequences of the phenomenon are far from clear. Indeed, the length of the Discussion highlights the nuance of the observation. The perceptual reports are almost certainly overestimates based on the predictability of the reports derived from the repetition of particular gaze shifts and limitations of specific stimuli. The skeptic can wonder whether the effects will be as "considerable and highly consistent" (line 232) if saccades of unpredictable amplitude and direction are made or if mixtures of spatial frequencies are judged. The highly-constrained and artificial testing conditions invite questions about the relevance for real-world perception.

This comment raises important questions regarding ecological validity, generality, and the interpretation of the experimental effects. We revised the manuscript extensively to address these concerns.

First, our use of controlled stimuli and instructed saccades was a deliberate choice aimed at isolating and testing specific theoretical predictions. The experiments in Figs. 2–3 and 5 were designed to provide an unambiguous demonstration that post-saccadic perception follows the spatial information redistributed by saccade-induced transients. The predictability of saccade amplitude and stimulus content in these experiments should therefore be viewed as a methodological strength for hypothesis testing, not as a limitation of the underlying mechanism. Importantly, the mechanism itself is not restricted to a particular saccade amplitude or stimulus class: the spatial information conveyed by saccade transients varies continuously with saccade amplitude and with the spatial-frequency content of the scene.

Second, to directly address the concern that the reported effects might not generalize to more natural viewing conditions, we added new experiments and analyses in the revised manuscript. In one experiment, saccade amplitude and direction varied unpredictably across trials, and the stimuli contained broadband spatial-frequency content rather than isolated gratings (Supplementary Fig. S5). In a second experiment, we tested perception using hybrid images composed of low- and high-frequency components of real-world scenes (Fig. 4). In both cases, the results were fully consistent with our predictions, demonstrating that the effect persists under less constrained and more naturalistic conditions.

Finally, we revised the Discussion (only ∼1,500 words in length) to more clearly explain the logic of the experiments, explicitly address relevance for real-world perception, and better articulate the emerging hypotheses raised by these findings. We also streamlined and reorganized the text to improve clarity and readability while retaining the contextualization necessary to situate the results within the existing literature.

Together, these additions show that the reported perceptual effects are not an artifact of constrained laboratory conditions, but reflect a general property of how saccade-induced transients shape visual encoding during natural vision.

The discrepancy between the predicted 300% difference in perception and the observed 37% difference (line 264) reinforces this query.

The apparent discrepancy reflects a misunderstanding arising from a paragraph in the Discussion that was not sufficiently clear.

The ∼300% value refers to a change in the *input power* on the retina after weighting and integrating the saccade-induced signal with a parameter-free *linear filter* taken directly from the neurophysiological literature. This model was intended to illustrate the *qualitative* consequences of saccade transients, not to provide a *quantitative* prediction of visual performance. A discrepancy between changes in retinal input power and the magnitude of the perceptual effect is expected, because both brightness and perceived contrast are related to stimulus strength via a compressive, nonlinear relationships and are further shaped by normalization mechanisms.

To avoid any ambiguity, in the revised manuscript we no longer refer to the linear-filter output as a "perceptual prediction." Instead, we now incorporate a standard compressive nonlinearity into the model so that it can be used to generate quantitative predictions of performance. As shown in the revised Fig. 2d, this modification brings model responses into close agreement with the empirical data. We rewrote the corresponding paragraph in the Discussion to eliminate possible misunderstandings (second paragraph of page 12).

Also, if this manner of improving visibility mattered in natural vision, observers should control saccade amplitudes in order to improve visibility as predicted by the authors' framework. Do they?

This is an interesting hypothesis arising from our study. Since saccade amplitude acts as a perceptual spatial filter, saccade planning may incorporate consideration of the spatial information provided by saccade-induced transients. Importantly, the perceptual enhancements we report occur irrespective of whether they are actively sought by the oculomotor system: foveation may well dominate saccade planning without diminishing the significance of the phenomenon we describe.

We expanded the Discussion on page 15 to elaborate on this possibility, and we note that motivating an exploration of this possibility is one of the broader implications of the work. Because amplitude-dependent changes in post-saccadic visibility were not recognized prior to this work, no previous study has directly examined whether scale selection and spatial selection interact in saccade planning. Testing this hypothesis will require carefully designed experiments to disentangle the contributions of factors. Moreover, under natural viewing, these processes may operate synergistically given the intrinsic coupling between object size and the spatial frequencies relevant for its analysis.

Nonetheless, several existing observations are consistent with the broader idea that saccade control may serve functional roles beyond foveation. First, converging evidence indicates that visual processing is not suspended during saccades and that the visual system continues to accrue information from retinal input during these movements [1–5]. Second, saccades occur in species without a fovea, supporting the view that their function is not limited to

foveation [6, 7]. Third, saccade amplitude is modulated by stimulus characteristics and task demands in ways compatible with a role of spatial scale: for example, saccade amplitude increases when observers search for low–spatial-frequency targets in natural scenes [8], and viewing low-pass filtered images yields larger saccades than viewing high-pass filtered images [9–11]. Finally, conditions associated with reduced foveal function or reduced acuity are often accompanied by increases in saccade amplitude, as observed in macular degeneration [12] and in mice reared under conditions—such as dark rearing or monocular deprivation—that reduce acuity [6, 13].

The relevant text is on lines 364–380 of the revised Discussion. In the revised text, we also emphasize that this is a hypothesis motivated by the present results, not a conclusion of our study.

Also, in real-world terms, what objects or parts of objects have the spatial frequencies used for testing, and how often do humans make 0.4, 1.0, and 6.0 degree saccades in viewing them?

As mentioned in our response to a previous point, we selected specific spatial frequencies and saccade amplitudes to provide an unambiguous test of the theoretical predictions. Natural scenes exhibit a $\sim 1/k^2$ falloff in power, so low spatial frequencies—such as those used in these studies—are prevalent during natural vision and convey information about the coarse, global structure of the visual scene (the overall layout of objects and surfaces). These are precisely the spatial components most strongly affected by saccade transients.

The saccade amplitudes used in these experiments (0.4°, 1°, and 6°) are also common in everyday viewing. Saccades smaller than 1° are predominant during high-acuity inspection and fine-detail analysis (*e.g.*, refs. 14–16), whereas saccades of 1° and above occur routinely during scene exploration, visual search, and reading [17].

We added text to the revised manuscript to explicitly address these points (page 17, lines 407–409; page 19, lines 455–458). And as mentioned above, we now add additional studies to demonstrate that our findings are not restricted to specific spatial frequencies and saccade predictability.

(2) The authors emphasize the image filtering in relation to saccade amplitude, but saccade velocity varies within amplitude. Indeed, in contrast to the assertion of the authors of "the tight relation between amplitude and speed" (line 94-95), other investigators have highlighted the variation in saccade velocity for a given amplitude. They describe variation of "saccade vigor" with task demands and other parameters. The authors' analysis should make specific predictions about perceptual variation across velocity for a given amplitude.

Our statement about the relation between saccade amplitude and speed refers to the well-established saccade main sequence: on average, larger saccades tend to have higher peak velocity, so that saccade duration increases only moderately with amplitude. As explained

in Mostofi et al. (2020), this relation is the primary factor determining how the power spectra of saccade-induced transients change with amplitude, as shown in Fig. 1d.

We fully agree that, for a given amplitude, saccade velocity can vary with task demands, motivation, or so-called "saccade vigor." However, our predictions are robust relative to these variations, because they follow directly from fundamental properties of saccade kinematics. Changes in vigor can alter the detailed temporal distribution of power (shifting some energy toward higher or lower temporal frequencies), but unless saccade kinematics deviate qualitatively from the main sequence (e.g., peak velocity decreasing with amplitude), they will have limited influence on the integrated power delivered within the temporal sensitivity band of the visual system. Consequently, larger-amplitude saccades are expected to yield stronger low–spatial-frequency transients than smaller ones, largely independent of variations in vigor.

To directly address this point empirically, we added a control analysis in Supplementary Fig. S2. For the saccades executed in the experiment of Fig. 3, we divided trials into slower- and faster-than-average groups at each amplitude and compared their spectral power distributions. As shown in Fig. S2b, the spectra for fast and slow saccades of a given amplitude are very similar, and in all cases a 6° saccade yields substantially stronger low–spatial-frequency transients than a 1° saccade. These results confirm that the perceptual effects reported here are robust to trial-to-trial variability in saccade velocity.

Minor comments

The description of methods at lines 139-141 can be explained clearer for less sophisticated readers.

We agree with the Reviewer and rewrote this section to be clearer and more self-contained, clarifying the steps from the retinal luminance time series to the spatiotemporal power spectrum and the subsequent integration within the temporal sensitivity band. While these procedures have been described in detail in our previous publications, including them here makes the materials easier to follow. The revised description can be found in the second paragraph of page 6.

The description of methods at lines 146-147 is not as clear and explicit as could be. The free viewing condition is not included in the main methods.

We revised the text in both the Results and Methods sections to more explicitly describe the origin of the free-viewing data and the purpose of this comparison (Results: lines 144-147; Methods: lines 528-531).

Because the rationale for our experiments was developed on the basis of transients generated by natural (non-instructed) saccades, in Figs. 2c and 5b we compare the power redistributions from the instructed saccades in our task with those obtained from a separate database of saccades recorded while observers viewed natural scenes. This comparison serves as a preliminary control to verify that the transient signatures of saccades in our experimental

paradigm are not qualitatively altered by task constraints. As shown in Figs. 2c and 5b, instructed and free-viewing saccades of comparable amplitudes yield very similar power distributions.

The rationale for reporting the 0.4 degree saccade data later in the manuscript was not clear. The busy reader would appreciate more brevity. The skeptical reader would like to see how consistently participants produced 0.4 degree saccades.

We revised this section to clarify the rationale and importance of the 0.4° saccade experiment (lines 224-236).

This experiment provides a particularly stringent test of the theory because, with the specific spatial frequencies used here, a small reduction in saccade amplitude (from 1° to 0.4°) is predicted to *reverse* the perceptual bias observed in Fig. 3 (*i.e.*, favor the higher rather than the lower spatial-frequency component).

This reversal follows directly from the amplitude dependence of the whitening range (Fig. 1d): for the smaller saccade, the Reference falls within the whitening band and thus elicits a weaker transient, altering the relative transient strength that drives visibility. Observing the predicted reversal therefore strengthens the conclusion that post-saccadic reports track the spatial information conveyed by saccade-induced transients within the temporal sensitivity band.

We also revised the text to address the Reviewer's concern about whether observers reliably produced 0.4° saccades. Saccades in this amplitude range occur frequently during natural high-acuity tasks (*e.g.*, Intoy et al. [16]) and are elicted reliably under instruction (see, for example, Fig. 6 in Poletti et al. [18]). In this experiment, only $\sim 3\%$ of the trials were excluded because the cue failed to trigger a saccade, and Supplementary Fig. S4a now reports the measured distribution of saccade amplitudes. The relevant text is on page 19, lines 455–458.

References

[1] T. L. Watson and B. Krekelberg. The relationship between saccadic suppression and perceptual stability. *Curr. Biol.*, 19(12):1040–1043, 2009.

[2] R. Schweitzer and M. Rolfs. Intrasaccadic motion streaks jump-start gaze correction. *Sci. Adv.*, 7(30):eabf2218, 2021.

[3] M. Boi, M. Poletti, J. D. Victor, and M. Rucci. Consequences of the oculomotor cycle for the dynamics of perception. *Curr. Biol.*, 27:1–10, 2017.

[4] M. Panouillères, V. Gaveau, C. Socasau, C. Urquizar, and D. Pélisson. Brain processing of visual information during fast eye movements maintains motor performance. *PLoS ONE*, 8, 2013.

[5] M.A. García-Pérez and E. Peli. Intrasaccadic perception. *J. Neurosci.*, 21, 2001.

[6] L. Wang, M. Liu, M. A. Segraves, and J. Cang. Visual experience is required for the development of eye movement maps in the mouse superior colliculus. *J. Neurosci.*, 35, 2015.

[7] S. H. Zahler, D. E. Taylor, J. Y. Wong, J. M. Adams, and E. H. Feinberg. Superior colliculus drives stimulus-evoked directionally biased saccades and attempted head movements in head-fixed mice. *eLife*, 10, 2021.

[8] L. O. M. Rothkegel, H. H. Schutt, H. A. Trukenbrod, F. A. Wichmann, and R. Engbert. Searchers adjust their eye-movement dynamics to target characteristics in natural scenes. *Sci. Rep.*, 9:1635, 2019.

[9] H. Deubel and T. Elsner. Threshold perception and saccadic eye movements. *Biol. Cybern.*, 54, 1986.

[10] M.T. Groner. The evect of spatial frequency content on parameters of eye movements. *Psychol. Res.*, 72, 2008.

[11] A. Cajar, R. Engbert, and J. Laubrock. Spatial frequency processing in the central and peripheral visual field during scene viewing. *Vision Res.*, 127:186–197, 2016.

[12] W. Seiple, R. B. Rosen, and P. M. T. Garcia. Abnormal fixation in individuals with age-related macular degeneration when viewing an image of a face. *Optom. Vis. Sci.*, 90, 2013.

[13] J. M. Samonds, W. S. Geisler, and N. J. Priebe. Natural image and receptive field statistics predict saccade sizes. *Nat. Neurosci.*, 21, 2018.

[14] M. Poletti and M. Rucci. A compact field guide to the study of microsaccades: Challenges and functions. *Vision Res.*, 118:83–97, 2016.

[15] N. Shelchkova, C. Tang, and M. Poletti. Task-driven visual exploration at the foveal scale. *Proc. Natl. Acad. Sci. USA*, 116(12):5811–5818, 2019.

[16] J. Intoy, N. Mostofi, and M. Rucci. Fast and nonuniform dynamics of perisaccadic vision in the central fovea. *Proc. Natl. Acad. Sci. USA*, 118:e2101259118, 2021.

[17] E. Kowler. Eye movements: The past 25 years. *Vision Res.*, 51(13):1457–1483, 2011.

[18] M. Poletti, J. Intoy, and M. Rucci. Accuracy and precision of small saccades. *Sci. Rep.*, 10, 2020.

Reply to Reviewer #1

Evaluation The authors Li, Cox, Victor, and Rucci have done a good job in revising their manuscript, but I still have two concerns, the first being the most relevant.

Concerns 1. Extended time windows around the saccade. In my previous review I voiced the concern that the authors' results – and that is in principle also the case for previous studies – are influenced by the pre- and post-saccadic intervals of stationarity that they add to the saccadic waveform. Their responses did not alleviate my concern, but created the impression that they did not know what I meant, which is why I would like to demonstrate it below using my own attempt to reproduce their results (given that this part of their code has not been made publicly available). Briefly, I simulated two compressed-exponential saccade trajectories according to the main sequence and computed the luminance changes induced when they are made across 100 bandpass-filtered noise images (SFs: 0.01, 0.5, 3 cpd), then computed the resulting TF spectra and weighted them by Kelly's (1979) sensitivity function, please see the figure I attached to this review. In a first condition (solid lines, circles) I wrapped the saccade waveform in either 250-ms noise-free fixation periods to replicate the authors' results: Indeed, larger saccades increase the resolvable power at low SFs, but not at high SFs, which is exactly what the authors report. In a second condition (dashed lines, triangles) I used only the saccade waveform, without fixation periods, to make the above point: While resolvable power is still boosted at low SFs, at higher SFs larger saccades decrease power – quite drastically – as higher saccadic velocities shift the spectrum towards even higher TFs, leaving increasingly little in the resolvable range. This is what Castet, Jeanjean & Masson (2002) found: Despite the distributing effect of the kinematic profiles of saccades and the broadband effect of the flash (both arguments used to respond to my concerns) the high-SF stimulus remained unresolvable. Another fitting example would be the indiscriminability of high-speed object motion – whether during saccades or fixation – if these objects are composed of high SFs (Schweitzer & Rolfs, 2020). What restores power at high SFs, however, is a stable post-saccadic image, a highly efficient process known as post-saccadic masking (e.g., Duyck, Wexler, Castet, Collins, 2018; Schweitzer, Doering, Seel, Raisch, Rolfs, 2025). The authors wrote that "what temporal window is being analyzed ... does not confound saccades with fixations" and I still have to disagree. The current results stand because of the specific way spectral analysis and the experiment at hand was performed, that is, embedding the saccade in continuously present stimuli that enable masking of strictly saccade-induced transients.

We thank the Reviewer for clarifying this point. We now understand the concern more precisely: the issue is not whether fixational eye movements contaminated our estimates, but whether the conclusions drawn from our spectral analysis depend on the periods of stationary luminance on either side of the saccade and, if so, what temporal window is scientifically appropriate.

The Reviewer points out that the spectrum of the luminance modulation is influenced by these periods. We fully agree: because of their very nature, spectral analyses *always* depend on the surrounding context, whether this is explicitly considered or implicitly determined by the analysis itself (see point 2 below). Our inclusion of buffer periods of constant luminance was a deliberate choice, motivated by both biological and mathematical considerations, as detailed below. In response to the Reviewer's comment, we have revised the manuscript to clarify these reasons, expanded the description of the fundamental principles of spectral estimation on which our approach relies, and added a new analysis in Supplementary Fig. 1.

1. Saccades as state transitions. Conceptually, these stationary periods are an integral part of the phenomenon under study. In natural viewing, a saccade is not an isolated phenomenon; it is a movement that starts and ends with zero velocity. On the retina, this movement yields a state transition in the visual input: it changes luminance from one constant value to another via specific dynamics. The surrounding periods of stationary luminance are therefore not an artificial addition, but part of the natural context in which saccades occur. In this study, we are interested in understanding the consequences of saccades as natural transitions in retinal input.

2. Computational necessity: avoiding spectral artifacts. Considering the intra-saccadic luminance modulation in isolation, without flanking stationary periods, introduces two classes of artifacts. The first is spectral leakage. Isolating the intra-saccadic segment is equivalent to multiplying the retinal signal by a narrow rectangular observation window. In the frequency domain, this multiplication corresponds to convolution with a broad sinc function, which spreads power across frequencies and thereby generates spectral leakage.

The second artifact is the introduction of spurious frequency components. This occurs because the DFT assumes periodic repetition of the analyzed segment[1], so analyzing a window containing only the intra-saccadic transient is equivalent to estimating the spectrum of an artificial waveform in which the motion segment repeats end-to-end indefinitely—essentially a never-ending, periodically discontinuous saccade. The discontinuities introduced at the boundaries of this repeated waveform generate frequency components that are not present in the original transient.

Thus, analysis of the isolated intra-saccadic modulation without stationary periods does not provide a more faithful estimate of the retinal input generated by the saccade. Rather, it analyzes an artificial signal and introduces distortions that obscure the actual luminance transition generated by the saccade. By flanking the saccade transient with buffers of constant luminance and applying windowing procedures to handle boundary discontinuities, as we do in the manuscript, we minimize both types of artifact and preserve the spectral content of the saccade transient itself.

[1] The DFT, by definition, provides the Fourier-series coefficients of the periodic repetition of the analyzed segment.

3. Functional interpretation. Critically, inclusion of the constant-luminance periods before and after a saccade is essential for a functional interpretation of the spectral analysis. The purpose of the spectral analyses in this manuscript is to enable direct estimation of the spatial information that a saccade makes available to the visual system. Each point in the maps of Fig. S1d is proportional to the strength of the response—both its energy and its peak amplitude—elicited in a linear filter tuned to the corresponding spatial and temporal frequencies when moving across a white-noise field. Integration across frequencies thus enables prediction of how the visual system would respond to the saccade. Our study builds on these predictions.

The analysis in Fig. 1 of this letter clarifies this point and reinforces the appropriateness of our spectral estimation method. Here, we estimate the strength of the response of a simple neural model (a linear filter) moving across a grating with a saccade-like trajectory (modeled as a sigmoid). In panel A, we perform a direct calculation: we convolve the filter with the spatiotemporal input and then compute response strength as the integral of the squared filter output. In panel B, following the approach used in the manuscript, we first estimate the spatiotemporal power spectrum of the visual input and then integrate spectral power over temporal frequencies weighted by the filter sensitivity. Critically, the spectral analysis closely matches the actual response of the filter only when the spectrum is estimated using our approach (Fig. 1B, blue lines, compared with the circles replotted from panel A). In contrast, when the spectra are computed using only the intra-saccadic segment, the calculation fails (Fig. 1B, green dashed lines) for the computational reasons described above. Notably, as mentioned by the Reviewer, this latter analysis predicts a reduction in response at high spatial frequencies, but this reduction is an artifactual consequence of the spectral procedure; it is not present in the filter response.

Fig. 1C shows a more direct example of this principle by predicting the responses of standard L-NL models of magnocellular retinal ganglion cells (*i.e.*, rectified linear spatiotemporal filters) during saccades across natural scenes. As predicted by our spectral analysis, responses increase with saccade amplitude only while the neuron's preferred spatial frequency (k) remains within the whitening region of the saccade, where the spectral whitening induced by the saccade counterbalances the $1/k^2$ falloff of natural scenes. This is the same information conveyed by our spectral maps, expressed here in terms of neural responses. The close correspondence between spectral power and filter output depends on preserving the stationary periods, as our long buffers ensure that the analysis window fully captures the saccade transient that drives these responses.

Fig. 1C further clarifies that, because these filters are causal, shortening the fixation interval would not alter their responses once activity has settled (after about 50 ms). In contrast, shortening the observation window in the spectral analysis so that it includes only the transient causes the estimate to deviate from the filter responses because of the DFT-related artifacts discussed above.

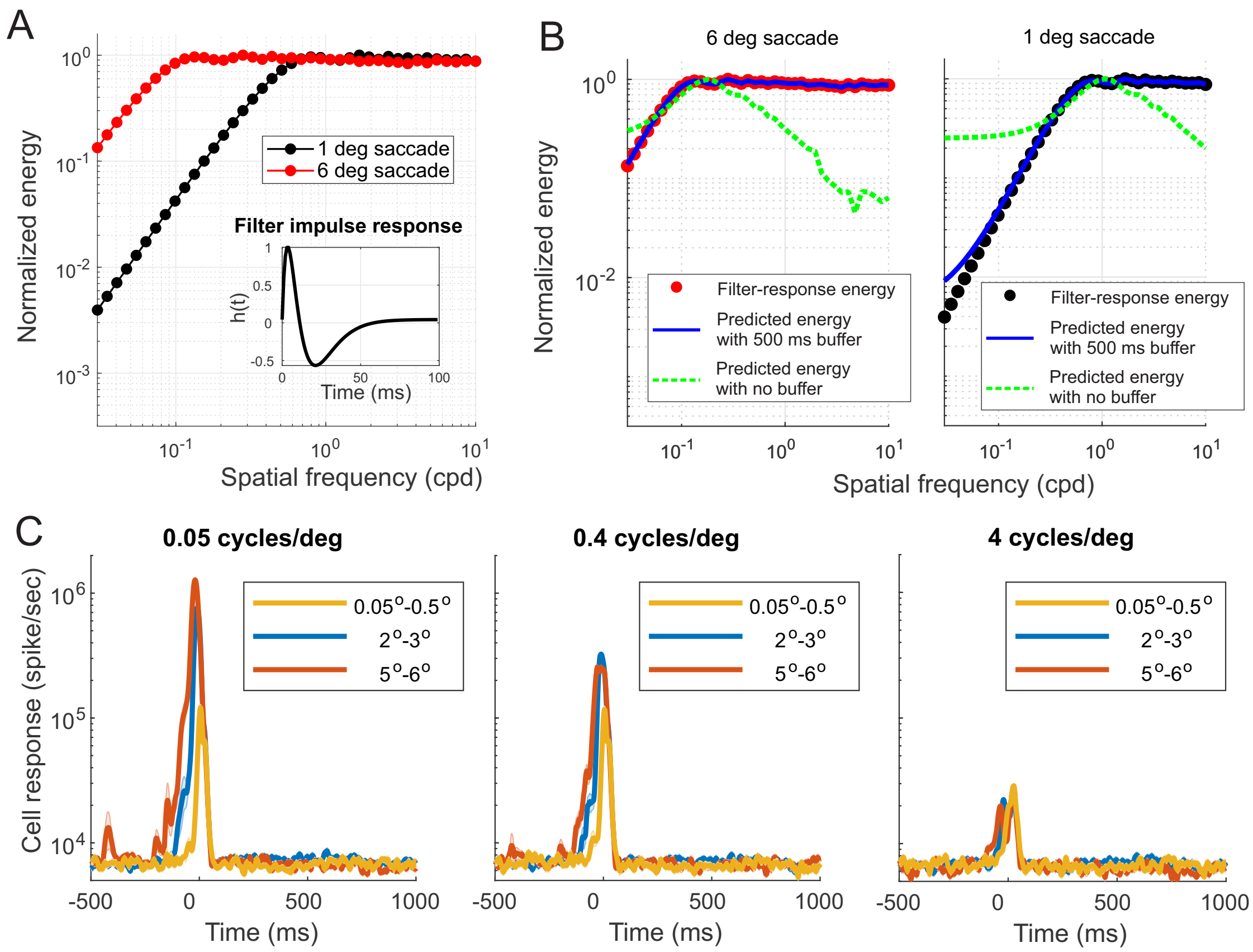

Figure 1: **Responses of linear neural filters to saccades and their prediction from spectral analysis.** (**A**) Response energy of a causal linear temporal filter as a function of stimulus spatial frequency for two saccade amplitudes (here modeled as sigmoidal functions). The larger saccade yields a stronger response at low spatial frequencies, as predicted from our spectral analyses. Note the close match to Fig. 1D in the manuscript. (**B**) Comparison between the response of a causal linear filter and the predictions obtained from spectral analysis. Circles show the filter-response energy measured directly in the time domain for a 6° saccade (red, left) and a 1° saccade (black, right). The blue line shows the spectral energy obtained with our method, which closely matches the filter response across spatial frequency (circles; the same curves shown in panel A, replotted here for comparison). The green dashed line shows the spectral energy obtained when the analysis is restricted to the intra-saccadic segment. This latter procedure incorrectly predicts a reduction in the filter response at high spatial frequencies, which arises from the spectral artifacts introduced by the truncated window. (**C**) Responses of standard L-NL models of magnocellular retinal ganglion cells during saccades on natural scenes. As predicted by our method of spectral analysis, responses increase with saccade amplitude (different colored lines) only while the neuron's preferred spatial frequency (indicated above each subpanel) remains within the whitening region of the saccade. Here, the attenuation in the responses with increasing spatial frequency is determined by the $1/k^2$ spectrum of natural scenes. The Matlab scripts used to generate panels **A–B** are provided with this submission.

This is a limitation to the interpretation of the results that needs to be clarified.

We respectfully disagree that our approach limits interpretation. As explained above, our method ensures that the spectral analysis remains a valid proxy for the spatial information actually extracted by the early visual system, which is the quantity we seek to represent. Our approach is therefore not an arbitrary analytical choice, but the appropriate way to estimate the spectrum of a finite transient.

Even better, of course, would be a direct test to see whether the current results would hold when accounting for the pre- and post-saccadic period. That is, I have no doubt that the enhancement of low SFs at larger saccades holds but I would really like to see a version of their spectral analysis using strictly saccadic windows, believing that this adds value and novelty to the analyses presented already in previous publications.

Figure S1E in the revised manuscript reports the spectral estimates obtained with the shorter observation windows requested by the Reviewer. The results remain qualitatively similar: in particular, the main finding of our study—the enhancement at low spatial frequencies with increasing saccade amplitude—is preserved. However, as explained above, these spectra do not provide a more faithful characterization of the saccade transient; rather, they introduce spectral artifacts because they correspond to artificial waveforms with limited biological interpretation. We have revised both the Methods and the Discussion to better explain why the approach used in the main manuscript is appropriate and how it relates to the responses of causal linear models.

2. The relationship to saccadic suppression. I believe that the authors have proposed an interesting mechanism relating their findings with classic findings of saccadic suppression. I believe it would be a good idea to include the additional figure in the manuscript – it is not distracting as it makes a specific prediction about the relevance of the results at hand that may be critically evaluated in future experiments.

We thank the Reviewer for this suggestion. We agree that the figure makes a testable prediction and have included it in the revised manuscript as Supplementary Figure S7.

I have two points to make though. First, Figure 1A is erroneous – as is Figure S1d (formerly Figure 1f) where the panels are now switched rendering the assignment large/small saccade incorrect – as there seems to have happened a switch-up between 1-deg and 6-deg saccades.

The labels of the panels in Figure S1d were indeed inadvertently swapped. We corrected the figure.

Second, there is a logical inconsistency in the argument. The authors derive their predictions from a case where saccades spatiotemporally redistribute SF-power of natural scenes, whereas most saccadic-suppression experiments have taken great care to remove background structure by using a Ganzfeld-like setup. Notably, despite a great reduction in suppression strength,

SF-selectivity persists even in near-total darkness (e.g., Burr, Holt, Johnstone, & Ross, 1982). How would these findings be accounted for in the proposed framework? Also, how is the given explanation compatible with “the absence of suppression observed for simulated—but not for real—saccades across uniform backgrounds or in complete darkness”? If anything, the mechanism the authors propose is visual-only and should be similar for real and simulated saccades - similar to what Idrees et al. (2020) found, but always requiring a structured background.

The Reviewer’s comment made us realize that the paragraph dealing with saccadic suppression, particularly its last sentence, which had been added in response to a request from Reviewer 2, was ambiguous and could be interpreted more broadly than intended. Our proposal was meant only as a possible contribution to the visually driven retinal component of suppression when a structured background is present, not as a general account of the spatial-frequency selectivity of saccadic suppression across all viewing conditions.

We fully agree with the Reviewer that the mechanism we propose is purely visual and would not apply under conditions in which visually driven background transients are absent or greatly reduced. We revised the paragraph accordingly to remove this ambiguity and to clarify that, under such conditions, suppression must depend on other factors, including extra-retinal signals associated with saccades.

(Remarks on code availability): The code for the model reported in this paper is available, but it does not generate figures, only a mat-file that comes without metadata. The code used for spectral analysis is unfortunately not available.

We have updated the code to include plotting routines and the spectral analyses. We have also included the Matlab demonstration scripts developed in response to the present review, because they make the rationale for the analysis methods used in the manuscript fully explicit.

Reply to Reviewer #3

In the revised manuscript the authors have addresses very effectively the issues raised in review of the original submission. Textual and graphic clarification (e.g., Fig 1e) and experimental extension substantiate the phenomena and justify publication in a Nature journal.

A few minor comments: (a) Discussing whether this effect on visibility can be incorporated into saccade planning, the authors include arguments derived from findings in mice. The logical relevance of these elements of the argument is unclear and seems to this reviewer even irrelevant.

The findings in mice suggest that altered spatial-frequency input can be associated with changes in saccade amplitude, implying that the frequency content of the visual environment may influence saccade amplitude. However, we agree that this point is somewhat tangential here, and we have therefore shortened the paragraph (lines 364–377) to focus on evidence more directly relevant to the proposed link between saccade transients and saccade planning.

(b) Figure 1c – The sequence of images in round apertures seem to relate to the image in 1a but not exactly. Seven images are (somehow) mapped onto 5 fixations. This potential confusion can be resolved by re-drawing or explaining.

The images were simply the input at a certain time with the same interval (every 250 ms) and not necessarily the fixations. We refined the panel and caption to better clarify this visualization.

(c) Figure 1d – The legend refers to "... $\pm$ one standard deviation (shaded regions)", but such shaded regions are not obvious in the plot.

We changed the shading in Figure 1d to make the $\pm$ one standard deviation regions more clearly visible.

(d) Line 750 – "evect" should be "effect"

Thanks for noticing this. We fixed the typo.

Reply to Reviewer #1

I thank the authors for their efforts to clarify their choices, which have now become very clear. While I still disagree with some of their views (see minor comment below), I see no reason to further delay the publication of this substantial paper and congratulate the authors for their good work.

Minor comment: I am fully aware of the artifacts that arise when looking at isolated saccadic or saccade-like periods, and therefore also understand why the authors have deliberately created an experimental paradigm that deliberately avoided them by adding long stimulus ramps. However, only because spectral analysis may be unsuited to estimate very short, strictly saccadic transients does not mean that these considerations should be excluded in an a-priori manner. That is, the visual system shows very predictable responses to spectral splatter. For instance, temporal frequencies up to 200 Hz can be accurately discriminated when embedded in brief temporal windows, whereas prolonging those temporal windows logically lowers thresholds to more reasonable values around 50 Hz. Without a doubt, the reported results are valid in natural viewing (which by definition always includes pre- and post-saccadic buffer periods) but high spectral power in high spatial frequencies during saccadic speeds may still seem highly unlikely for some workers in the field, if it were not for the introduced buffer periods. Just to mention one example, Gabor patches moving at saccadic speeds or beyond (even considering the spectral splatter that occurs as the stimulus rapidly enters and exits the receptive field) become invisible as a mere consequence of human temporal sensitivity (Rolfs, Schweitzer, Castet, Watson, & Ohl, 2025, Nat Comm). That is, even if pre- and post-saccadic endpoints were presented and saccadic velocity profiles were used. The same has been shown for motion smear, where the high spatial-frequency domain only regain relevance for the perceptual judgment as the duration of the post-saccadic window of stability is increased (Schweitzer, Doering, Seel, Raisch, & Rolfs, 2025, Psych Rev). In principle, these empirical results are now supported by the additional data points in Fig S1. Whether the authors want to explicitly mention them in their manuscript – in order to relate their results to a wider set of experimental settings or the critical choice of temporal windows – is of course up to them.

We thank the Reviewer for the positive assessment of our work and for this final suggestion. We have followed the Reviewer's suggestion and added citations to the two articles mentioned in the comment. We mention these studies in a new paragraph in the Discussion as examples of related paradigms in which analyses of the spatiotemporal input on the retina may also be informative (lines 357–360).

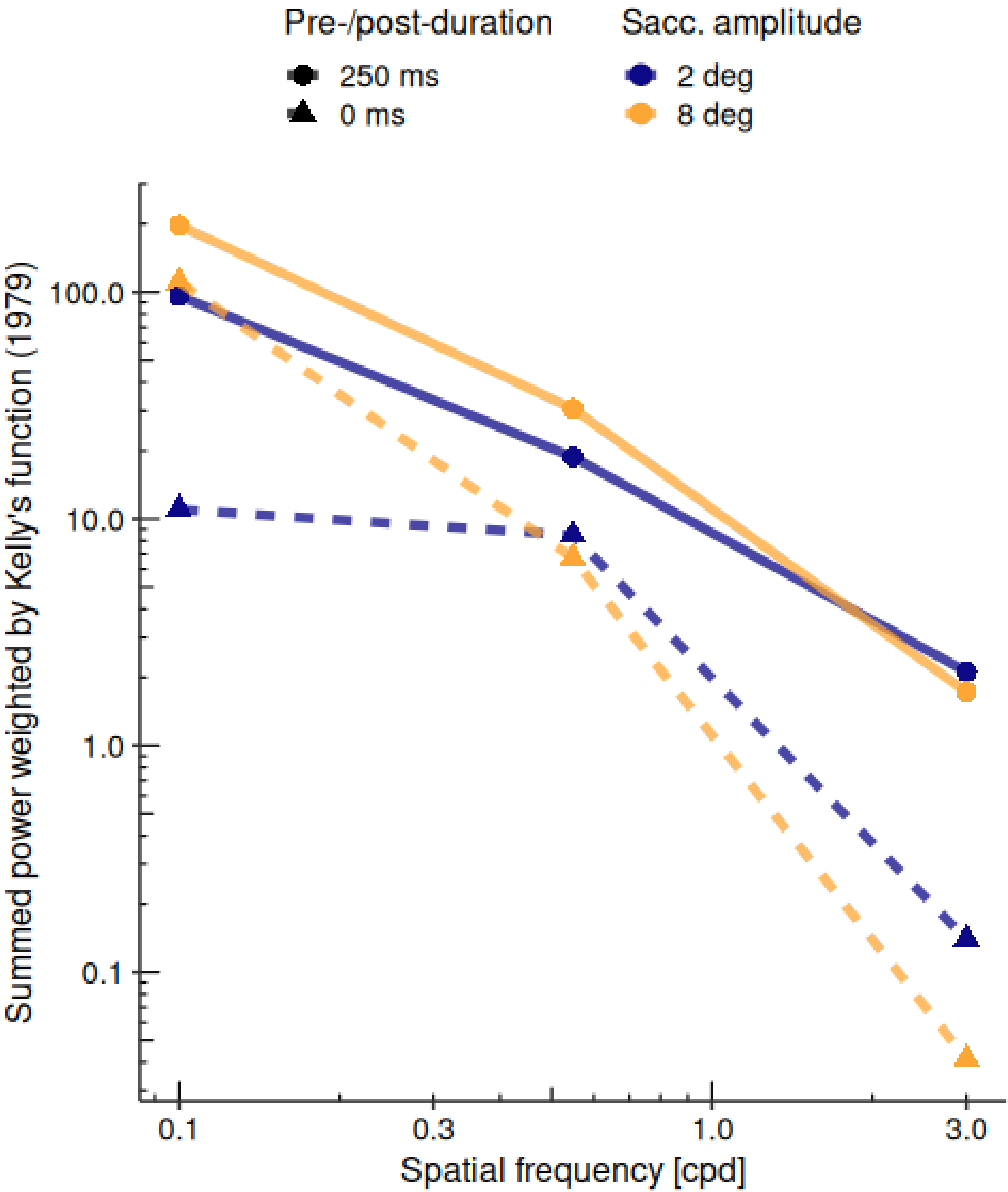

Pre-/post-duration
250 ms
0 ms
Sacc. amplitude
2 deg
8 deg
Summed power weighted by Kelly's function (1979)
100.0
10.0
1.0
0.1
0.1
0.3
1.0
3.0
Spatial frequency [cpd]
